# Porous Hybrid Soft Actuators From Liquid Crystal Networks and Lyotropic Chromonic Liquid Crystal Templated Hydrogels

**DOI:** 10.1002/adma.202516677

**Published:** 2026-02-15

**Authors:** Ramón Santiago Herrera Restrepo, Irving Hafed Tejedor García, Matthew Gene Scarfo, Negin Bouzari, Negar Rajabi, Olga Bantysh, Joel Torres‐Andrés, Christopher W. V. James, Maria Guix, Amirreza Aghakhani, Jordi Ignés‐Mullol, Salvador Pané, Josep Puigmartí‐Luis, Hamed Shahsavan

**Affiliations:** ^1^ Departament de Ciència de Materials i Química Física, Institut de Química Teòrica i Computacional (IQTC) Universitat de Barcelona Barcelona Spain; ^2^ Department of Chemical Engineering University of Waterloo Waterloo Ontario Canada; ^3^ Departament de Ciència de Materials i Química Física Institute of Nanoscience and Nanotechnology (IN2UB) Universitat de Barcelona Barcelona Spain; ^4^ Institute of Biomaterials and Biomolecular Systems (IBBS) University of Stuttgart Stuttgart Germany; ^5^ Multi‐Scale Robotics Lab Institute of Robotics and Intelligent Systems ETH Zurich Zurich Switzerland; ^6^ Catalan Institution for Research and Advanced Studies (ICREA) Barcelona Spain; ^7^ Department of Chemical Engineering Waterloo Institute for Nanotechnology Institute for Polymer Research University of Waterloo Waterloo Ontario Canada

**Keywords:** bilayers, hydrogels, liquid crystals networks, lyotropic chromonic liquid crystal, porosity

## Abstract

Liquid crystal networks (LCNs) have shown great utility in soft robotics as artificial muscles. Yet, their potential in biomedical applications, such as drug delivery, remains largely untapped. This can be partly attributed to LCNs’ limited compatibility with biological environments, and non‐porous microstructure and morphology. The current study focuses on developing actuators with improved porosity by creating constructs based on LCNs hybridized with liquid crystal hydrogels (LCHs). In our design, LCNs provide mechanical integrity and stimuli‐responsiveness, while LCHs introduce structural porosity and precise control over deformation. To manipulate LCHs’ microstructure and program the deformation of hybrid actuators, we used magnetically aligned lyotropic chromonic liquid crystals (LCLC) derived from disodium cromoglicate (DSCG) to template desired morphologies in acrylamide (AAM)‐based LCHs. Our results revealed that the integration of LCHs into LCNs dramatically increases the porosity of the construct. Interestingly, the distinct alignment and stimuli‐responsiveness of LCN and LCH layers can be leveraged to obtain complex programmable deformation. We believe that the inherent porosity and biocompatibility of LCHs can be used to expand the application of LCNs in therapeutic delivery and in enabling safer interaction with biological tissues, positioning them as promising materials for use in minimally invasive medical devices and adaptive implants.

## Graphical abstract

The graphic illustrates the design of a hybrid bilayer composed of a liquid crystal hydrogel (LCH) and a liquid crystal network (LCN). The figure highlights the magnetic alignment of the chromonic hydrogel and the thermal‐responsive actuation of the LCN along with the LCH when both layers are aligned or misaligned, enabling programmable, light‐driven deformations. This synergistic bilayer system demonstrates multi‐responsive actuation for advanced soft material applications.

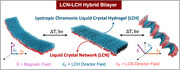

## 1 Introduction

Structurally hybrid soft materials offer great potential in developing adaptive and multifunctional actuators for applications in soft robotics [1, 2, 3]. Among a myriad of soft materials, liquid crystalline systems have been widely used as soft actuators, given the interesting, rich, yet complex physicochemical changes associated with the conformational change they experience in response to external stimulation. These behaviors are often rooted in their anisotropic molecular geometry, which at larger scales creates orientational or positional order and mesomorphic properties. The integration of liquid crystalline systems into elastic networks like liquid crystal elastomers and networks (LCNs) and liquid crystal hydrogels (LCHs) has proven to be an efficient approach to create soft actuators that reversibly change their shape when exposed to external stimuli in a highly controlled and programmable fashion. Polymerized liquid crystalline systems can be employed as artificial analogues of soft biological cells, tissues, and organisms, mimicking their flexibility and structural adaptability, and as such, showing potential in tissue engineering, drug delivery, and soft robotics [3, 4, 5].

While LCNs have been predominantly used as stimuli‐responsive actuators and artificial muscles in soft robotics, their potential as drug carrier systems is overlooked [6, 7, 8]. We attribute the limited applicability of LCNs in drug‐delivery systems to their typically less‐favored compatibility with water‐based systems, and more importantly, to their poor porosity. A recent work by Stepulane et al. synthesized lyotropic LCEs using polydimethylsiloxane (PDMS) and Pluronic F127 micelles and demonstrated their efficacy as drug carriers for Ibuprofen and Vancomycin. However, they did not report on shape change, wireless control, or even the remote activation of the system with external fields [9]. Furthermore, other works, such as Chen and coworkers’, have explored the design and fabrication of shape‐morphing polymer systems and demonstrated how these materials enable complex and reversible transformations in response to various external stimuli. Among the different systems discussed, liquid crystal elastomers (LCEs) stand out due to their intrinsic anisotropy and molecular alignment, which facilitate the nematic–isotropic phase transition and translate it into a macroscopic deformation. This coupling between molecular order and mechanical response enables continuous, programmable actuation with high repeatability and minimal mechanical fatigue [8]. Another recent example in drug delivery is the combination of siloxane liquid crystal polymers (LCPs) with polyvinylidene difluoride (PVDF) membranes to promote a well‐controlled on‐off drug release upon temperature increase. However, the biocompatibility and hydrophilicity of the resulting material were not systematically evaluated [10].

In this work, and inspired by previous reports, we attempt to integrate LCNs with biocompatible hydrogels (e.g., polyacrylamide (AAM)) to enhance their porosity and affinity with water [11]. We showcase that embedding lyotropic chromonic liquid crystal (LCLC) templates into the AAM hydrogel matrix, forming what we refer to as liquid crystal hydrogels (LCHs), enables precise tuning of their morphology and porosity [12, 13]. Previous work, including the report by Wang et al., used surface alignment for programming of hydrogel actuators templated from LCLCs [12]. While successful, this method bears an inherent limitation: the alignment of LCLC follows the alignment of the command surface. In a different approach, touchless and high‐fidelity manipulation of liquid crystalline orientational order was entertained and decoupled from the surface alignment using magnetic fields [14, 15, 16]. Therefore, the directed assembly and orientation of LCHs under external magnetic fields can be utilized as a robust tool for programming the shape change of LCNs integrated into them. For example, the integration of distinct yet complementary components (i.e., LCN and LCH) offers a novel platform to form hybrid structures capable of achieving programmed deformations with good biocompatibility and hydrophilicity, crucial for biomedical applications such as drug delivery. Furthermore, LCE materials and related hybrid systems have also shown significant potential in tissue engineering. Jiang et al. demonstrated that the mechanical compliance and reversible actuation of LCEs can create dynamic environments favorable for cell growth and tissue regeneration [6]. This highlights a promising future for LCEs and hybrid materials in the development of next‐generation biomedical and soft robotic systems. Although such applications have not yet been demonstrated, the findings reported here portray an opportunity to design new soft robotic systems with deterministic shape‐shifting and drug delivery capabilities [12, 17, 18].

## 2 Results and Discussion

### 2.1 LCHs From LCLCs

**FIGURE 1:**
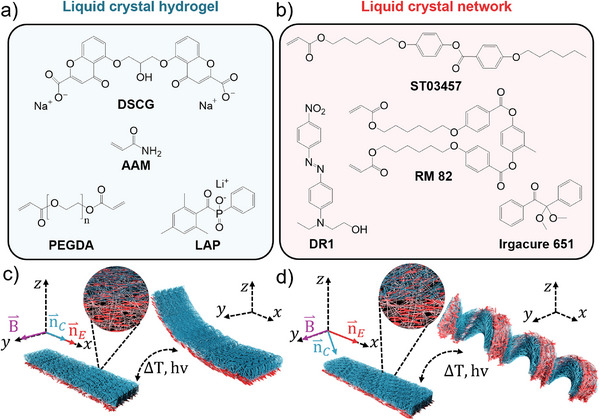
(a,b) are the molecular structures of the liquid crystal hydrogel (LCH) and liquid crystal elastomer network (LCN) components, respectively. **DSCG** = Disodium cromoglycate, **AAM** = acrylamide, **PEGDA** = polyethylene glycol diacrylate, **LAP** = lithium phenyl 2,4,6 trimethyl, **ST03457** = 4‐((6‐(acryloyloxy)hexyl)oxy)phenyl 4‐(hexyloxy)benzoate, **RM82** = 1,4‐Bis[4‐ (6‐acryloyloxyhexyloxy)benzoyloxy]‐2‐methylbenzene, **DR1** = Dispersed Red. (c,d) show schematic representations of the designed composite consisting of an LCN film (in red) and an LCH (blue). *B* ^⇀^ = magnetic field direction. 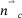 = LCH director field. 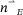
= LCN director field. ∆T = Temperature changes. hv = UV light irradiation.

The bilayer configuration combining an LCN and an LCH leverages the high porosity, biocompatibility, and programmable shape‐shifting capabilities arising from the distinct molecular alignment and morphology of each layer. Our design relies on two well‐established LCH and LCN systems made from disodium cromoglicate (DSCG) templated acrylamide hydrogels and acrylate‐based reactive mesogens, respectively (Figure 1a,b) [2, 13, 19, 20, 21, 22]. The deformation of LCNs can be tuned by the microstructure and morphology of the final AAM hydrogels templated from LCLC aggregates and aligned with magnetic fields before polymerization. When the director of the planar side of splay LCN and the director of LCLC are parallel, the actuator develops the expected bending (Figure 1c). In contrast, the presence of a misalignment between the director of the two layers causes the frustration of the pure bending of the splay LCN, developing a helical deformation (Figure 1d). In such a design, the synthesis of AAM hydrogel in the presence of a nematic LCLC phase is crucial to achieve the desired morphology, porosity, and shape‐change programming. To control the hydrogel microstructure and the final actuator's deformation, we utilized a template‐based method in which the anisotropy of nematic LCLCs directs the anisotropy of the LCH network [12, 13, 21].

For the LCH synthesis, we used highly biocompatible mixtures of DSCG within their nematic phase window (0.16 *≤ C_DSCG_ ≤* 0.26 g mL*^−^* ^1^) [13, 14]. DSCG presents a poor absorption of ultraviolet light (UV), making it suitable to be used with common photopolymerizable monomers, such as acrylates, and photoinitiators, such as lithium phenyl‐2,4,6‐trimethyl (LAP). In our initial trials, we explored different mixtures of DSCG and monomers, including N‐isopropyl acrylamide (NIPAM), methacrylic acid (MAA), and acrylamide (AAM) at different concentrations, finding that only mixtures containing AAM were able to form stable homogenous solutions. In contrast, when mixed with nematic DSCG solutions, aqueous solutions of other monomers underwent phase separation and formed solid‐like aggregates, hindering the polymerization of mixtures to uniform films. Afterward, polyethylene glycol diacrylate (PEGDA) was added to AAM/DSCG mixtures for photo‐crosslinking purposes. PEGDA was chosen for its superior biocompatibility, hydrophilicity, and enhanced tunability regarding its mechanical properties compared to other common crosslinkers, like N‐methylene bisacrylamide (BIS) [22]. As shown in Figure 1a, our final LCH formulation was based on DSCG as an LCLC template, AAM as the main polymer backbone, PEGDA as the crosslinker, and LAP as a photoinitator. Figure 1b shows the formulation of the shape‐shifting LCN layer in our hybrid actuator, composed of a bifunctional monomer as the cross‐linker (1,4‐Bis‐[4‐(6‐acryloyloxyhexyloxy)benzoyloxy]‐2‐methylbenzene or ST03457), a monofunctional monomer as the polymer backbone (4‐(6‐Acryloxy‐hex‐1‐yl‐oxy)phenyl 4‐(hexyloxy)benzoate or RM 82), Disperse Red 1 (DR1) as a photothermal heat generating dye, and 2,2‐dimethoxy‐2‐phenylacetophenone (Irgacure 651) as photoinitiator [2, 20].

**FIGURE 2:**
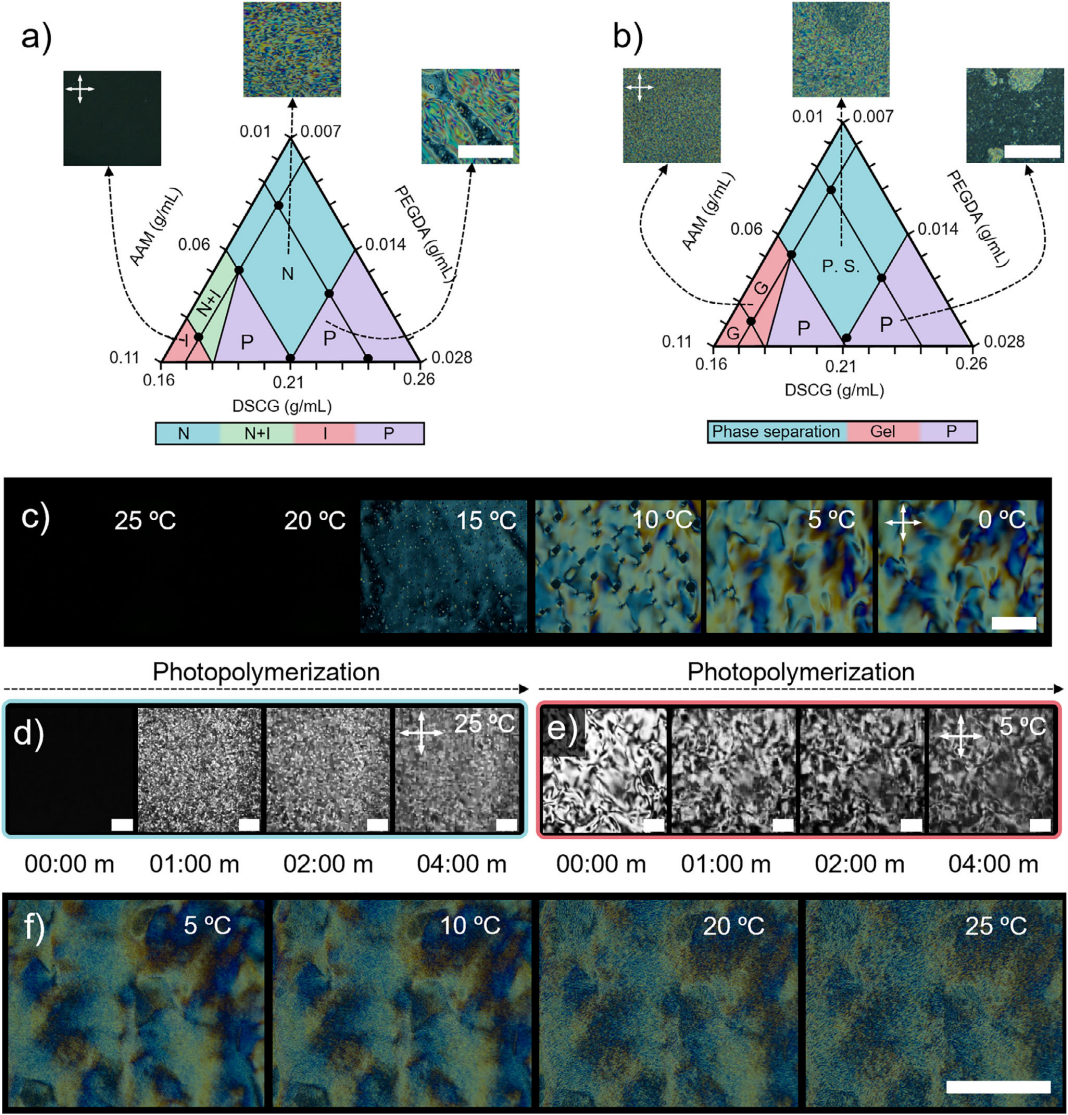
(a) Phase diagram for a mixture of acrylamide (AAM), polyethylene glycol di‐acrylamide (PEGDA), and disodium cromoglycate (DSCG) at different concentrations at room‐temperature. N stands for nematic phase, N+I for a coexistence phase between nematic and isotropic, I for isotropic phase, and P for the cases where a solid‐like phase precipitated. The inserts are representative POM images of each phase. Scale bar: 500 µm. (b) Phase diagram upon polymerization of the phases in (a), leading to the formation of a solid and homogeneous gel (Gel), and formation of a viscous and heterogeneous liquid phase (Phase separation). The insets are representative POM images of each photopolymerized gel. Scale bar: 500 µm. (c) Representative POM images of the temperature decrease effect of an isotropic phase at a concentration of 0.18 g mL*^−^* ^1^ DSCG, 0.09 g mL*^−^* ^1^ AAM, 0.007 g mL*^−^* ^1^ PEG, and 0.002 g mL*^−^* ^1^ LAP. Scale bar: 500 µm. (d,e) are time‐lapse POM images of the photopolymerization process carried out at 25°C and 5°C, respectively. (f) POM images sequence showing the temperature increase effect in a sample initially polymerized at 5°C. Scale bar: 500 µm.

Figure 2a and Figure S1a show the phase diagram of three‐component mixtures of DSCG, AAM, and PEGDA. To obtain the optimal composition of LCH precursor that would not adversely affect the nematic signature of DSCG at room‐temperature, we studied different concentrations of AAM and PEGDA. Samples were evaluated within a capillary cell (ca. 50 µm gap) under a polarized optical microscope (POM). The nematic signature of DSCG dispersions was preserved in mixtures with lower AAM concentrations (*C_AAM_ <* 0.06 g mL*^−^* ^1^). Mixtures with 0.06*< C_AAM_ <* 0.09 g mL*^−^* ^1^ showed nematic phase‐separated regions within continuous isotropic phases. Indeed, mixtures transformed to a fully isotropic state when *C_AAM_* was *>* 0.09 g mL*^−^* ^1^. In addition, mixtures were more susceptible to phase separation upon the addition of the PEGDA. Finally, nematic mixtures experienced precipitation of birefringent aggregates within isotropic continuous phases, even with slight increases in PEGDA concentration, especially in the case of *C_PEGDA_ >* 0.014 g mL*^−^* ^1^.

Figure 2b and Figure S1b show the phasic signature of hydrogels generated by polymerizing the mixtures shown in Figure 2a and Figure S1a (Note S1), respectively, with the addition of LAP as photoinitiator at room‐temperature. Mixtures with *C_AAM_ <* 0.06 g mL*^−^* ^1^ (typically viscous at pre‐polymerization) turned into extremely soft gels at post‐polymerization, showing a polydomain phase with smaller nematic domains when compared to their pre‐polymerized counterparts. Interestingly, isotropic mixtures with *C_AAM_ >* 0.09 g mL*^−^* ^1^ transitioned to nematic and exhibited micro‐phase separated nematic domains, most likely due to the decrease in the mobility and self‐assembly of DSCG‐rich solutions trapped within the hydrogel network meshes after polymerization stops. Mixtures with a high content of PEGDA, which showed precipitation within isotropic phases, polymerized to mostly isotropic heterogeneous and brittle hydrogels with sporadically dispersed solid aggregates. At this stage, we focused on mixtures of 0.18 g mL*^−^* ^1^ DSCG, 0.13 g mL*^−^* ^1^ AAM, 0.008 g mL*^−^* ^1^ PEG, and 0.009 g mL*^−^* ^1^ LAP as an optimal composition, yielding nematic and homogenous hydrogels.

All the nematic hydrogels display finer domains compared to their pre‐polymerized mixtures, which can be attributed to the increase in the thermal fluctuations typically caused by exothermic polymerization events. We postulate that the porosity and morphology of final hydrogels, which most likely depend on the size and shape of pre‐polymerized nematic domains, can be better controlled at lower temperatures. Our further phase study experiments at lower temperatures demonstrated an isotropic‐to‐nematic transition temperature of 15°C and a more stable nematic phase between 0°C and 15°C (Figure 2c). Differential scanning calorimetry (DSC) revealed a broad peak at low temperatures, corresponding to the observed nematic phase transition (Figure S2). When the sample was heated above room‐temperature, a second peak was observed, attributed to thermal polymerization and precipitate formation. The findings also indicated that adding a monomer to the nematic phase altered the heat of fusion and the transition temperature of the first peak, consistent with the loss of the nematic phase at high monomer concentrations.

**FIGURE 3:**
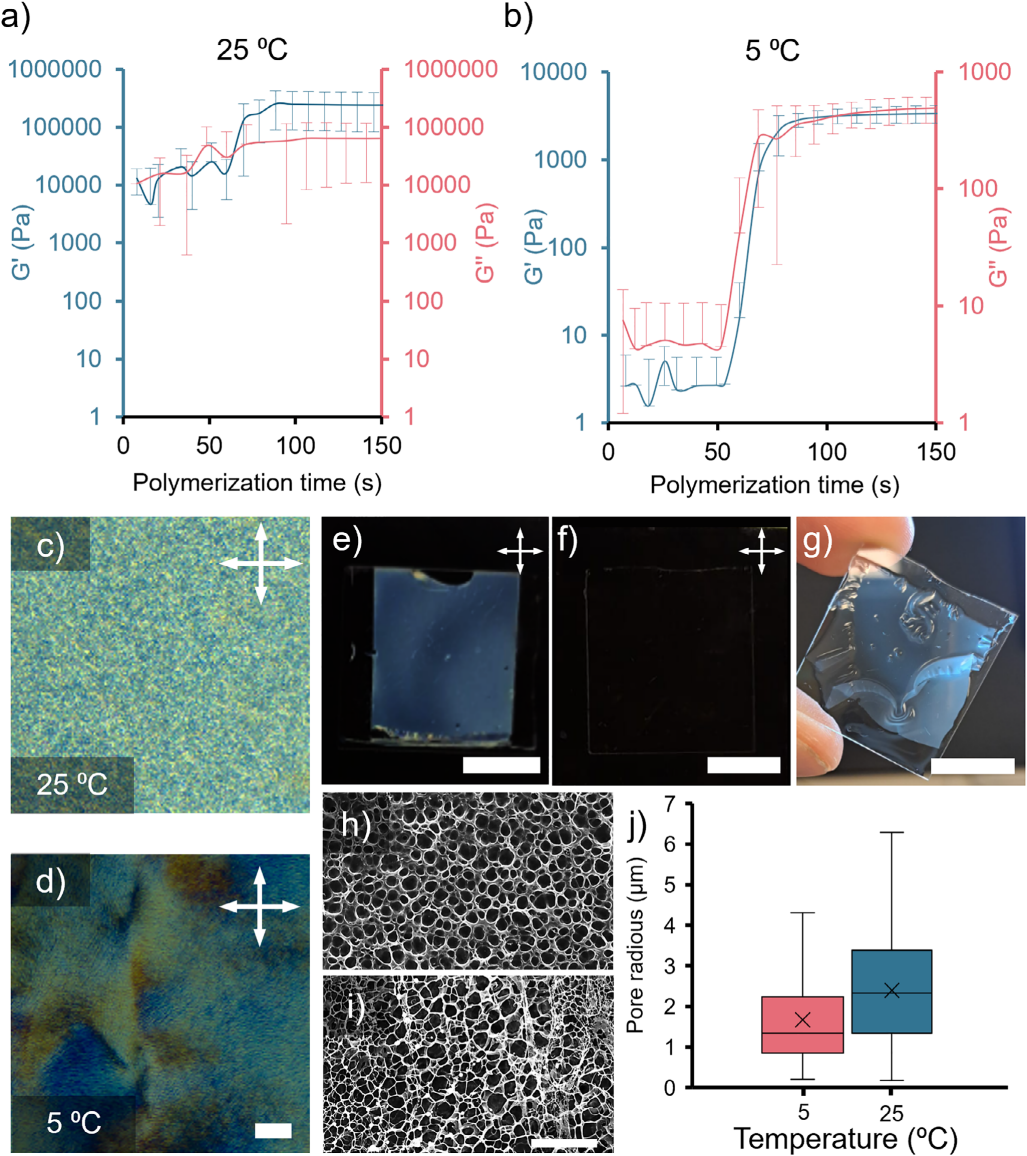
(a,b) are the photorheology characterization of the polymerization process of a mixture composed of 0.18 g mL^−1^ DSCG, 0.09 g mL^−1^ AAM, 0.007 g mL^−1^ PEG, and 0.002 g mL^−1^ LAP. Measurements were performed at a frequency of 1.6 Hz and a strain of 1% at 25°C and 5°C, respectively. We show the *G*´ (storage modulus) and *G*´´ (loss modulus) with their values on the left and right y axes, respectively. (c,d) are representative POM images of the samples polymerized at each studied temperature, 5°C and 25°C, respectively. Scale bar: 200 µm. Number of studied samples: 5. (e,f) are POM images of a sample before and after being rinsed with water to remove the DSCG template. Scale bar: 1 cm. g) Bright‐field image of the sample upon removal of DSCG after water rinse. Scale bar: 1 cm. (h,i) are representative SEM images of the obtained DSCG templated hydrogel after being polymerized at 5°C and 25°C, respectively. Scale bar: 50 µm. (j) Pore size radius evaluation of the images shown in (h,i).

To investigate the effect of temperature on the phasic behavior of hydrogels during and after polymerization, we monitored them under POM (Figure 2d–f). Isotropic mixtures at room‐temperature initially presented dark patterns that turned into birefringent bright mixtures under POM, just after 10 s of light exposure. This transformation can be considered as the beginning of the gelation process and the formation of tiny DSCG aggregates, which, after 1 min of exposure to light, progress into a fully frozen‐in state (Figure 2d). As will be discussed later in Figure 3, it is unlikely that the observed birefringence is the result of anisotropic chain growth during the polymerization for two reasons: (1) our precursors did not show a nematic mesophase at 25°C (Figure 2c), and (2) hydrogels made at 25°C did not show birefringence after washing of DSCG (Figure 3f). This means that we may have two dependent phenomena behind the appearance of birefringence during polymerization. As the polymerization reaction starts, the AAM monomers turn into growing chains with lower solubility. Growing chains and viscosity of the precursor upon polymerization limit the mobility of DSCG, favoring their aggregation to tiny birefringent domains. Isolation of these aggregates and their lack of mobility due to the constraints from the hydrogel network inhibit the formation of a global nematic mesophase with a longer range of orientational order required to form a nematic mesophase observed for samples polymerized at 5°C. After several minutes exposed to light, the samples’ opacity was altered, giving rise to secondary faded textures after 2 min (Figure 2e). The texture of these hydrogels was monitored as they were heated to 25°C (Figure 2f), revealing that at higher temperatures, darker secondary patterns appeared, while the initial nematic pattern persisted. These changes were attributed to the phase transition of the DSCG‐rich domains, which, although embedded in the hydrogel network, are not chemically bound to it. Therefore, we demonstrated that the nematic DSCG LCLC domains play a key role as templates for directing chain growth during polymerization. Subsequent removal of such a template yields hydrogels with anisotropic or tailored microstructure.

The change in DSCG concentration and its phasic behavior directly impacts the morphology and microstructure of the resulting hydrogels, determining their final mechanical properties and stability. To better understand the mechanical properties of precursors and polymerized hydrogels, we studied them with photorheology at room and low temperatures (Figure 3a,b; Figure S3 and Note S2). The precursor exhibited nonlinear viscosity at both high and low temperatures at the studied shear rates (0.1 to 1000 Hz), reminiscent of a shear‐thinning behavior (Figure S3a,b). Precursors tested at 5°C showed the same rheological behavior with lower shear stress and viscosity values (Figure S3a,b). Overall, viscosity decreased considerably as the shear rate increased for the precursor solutions. Upon sample photopolymerization of the samples, our results consistently showed that higher stress and viscosity levels increased and differed between the samples in the precursor solution and the polymerized gel (Figure 3a,b; Figure S3a,b). Here, the stress and viscosity at room and low temperatures increased by one and three orders of magnitude, respectively, after sample irradiation. This suggests that low temperature conditions resulted in a softer hydrogel due to the development of the nematic phase, which altered its internal structure. Since the reaction was performed at 5°C, the molecular mobility was reduced during photopolymerization as a consequence of the medium viscosity change, mediated by the nematic phase. This promoted the formation of a tighter packing and stronger interchain interactions. We observed consistently high complex viscosity values, indicating the integrity of hydrogel‐maintained structures and their elastic behavior within the linear viscoelastic region (LVR). A low‐strain LVR implies that the hydrogel is soft and easily deformable.

Photorheology experiments were also performed using time sweep measurements of the storage modulus (*G*′) and loss modulus (*G*″) at 1.6 Hz frequency and 1% strain. Tests were conducted on precursor formulations in their liquid state before UV exposure, and monitored in real time during photopolymerization under UV light (10 mW/cm^2^) at both room‐temperature (25°C) and low temperature (5°C) (Figure 3a,b). The results of photorheology measurements revealed the formation of softer hydrogels at lower temperatures, with storage and elastic moduli three orders of magnitude lower than the values obtained at room‐temperature. We also observed a displacement in the crossover time that increased when the process was taking place at low temperatures, suggesting differences in the polymerization reaction kinetics. Photopolymerization occurred at different rates depending on the exposure temperature, proceeding more rapidly at 25°C. This is likely due to the phase‐dependent molecular organization of the precursor, with the sample being in the isotropic phase at 25°C and in the nematic phase at 5°C. In both cases, gelation occurred within a few seconds of UV exposure, indicating the fast kinetics of network formation. We found that at low temperatures, the process was slower than at room‐temperature, indicating differences in the kinetic characteristics of the reaction, and thus, differences in the final gel. At low temperature, a longer polymerization time was observed with a smaller slope value in comparison to room‐temperature conditions (Figure S4). The results obtained can explain the different gel microstructures produced at high and low temperatures, as shown in the following sections. Further details and discussion of the storage (*G*′′) and loss (*G*″) moduli ratio (tan δ) can be found in Figure S5 and Note S3, where we discuss how the presence of the nematic phase altered the kinetics of the polymerization and the mechanical properties of gels. Our results show that the gelation onset is dominated by the photo‐initiation, but post‐gelation viscoelastic evolution is temperature dependent, leading to gels produced at lower temperature being softer than those produced at room‐temperature.

While hydrogels made of AAM and PEGDA were typically transparent, hydrogels containing DSCG were opaque, even after DSCG had been washed out (Figure 3c,d). We attribute this opacity to the larger pore size of the hydrogel networks formed in the presence of DSCG aggregates and DSCG‐rich phase‐separated domains. Indeed, the birefringence of LCH was eliminated after washing DSCG off the network. Micrographs obtained from scanning electron microscopy (SEM) confirmed the presence of large pores ranging from tens of nanometers to micrometers (Figure 3h,i), which may be the cause of light scattering and opacity in the samples. SEM images revealed that pore size varied with temperature, showing notably larger pores when the reaction was conducted at room‐temperature (Figure 3j). We also conducted Brunauer–Emmett–Teller (BET) surface area measurements (Note S4 and Figure S6). The single‐point surface area analysis revealed that LCH samples have much larger specific surface area (∼ 17m^2^/g) than LCN samples (∼ 0.9 m^2^/g). BET surface area of LCH–LCN bilayer is ∼ 5 m^2^/g, which is smaller than that of LCH, but still much larger than the surface area of LCNs. All these results suggest that the LCLC nematic character of DSCG influences the kinetics of the reaction, and the resulting hydrogel microstructure formed at room and low temperatures, with the latter showing potentially useful mechanical properties for actuation control. Therefore, the rest of the experiments were conducted on samples prepared at 5°C.

### 2.2 LCH Anisotropy From LCLC Templates

**FIGURE 4:**
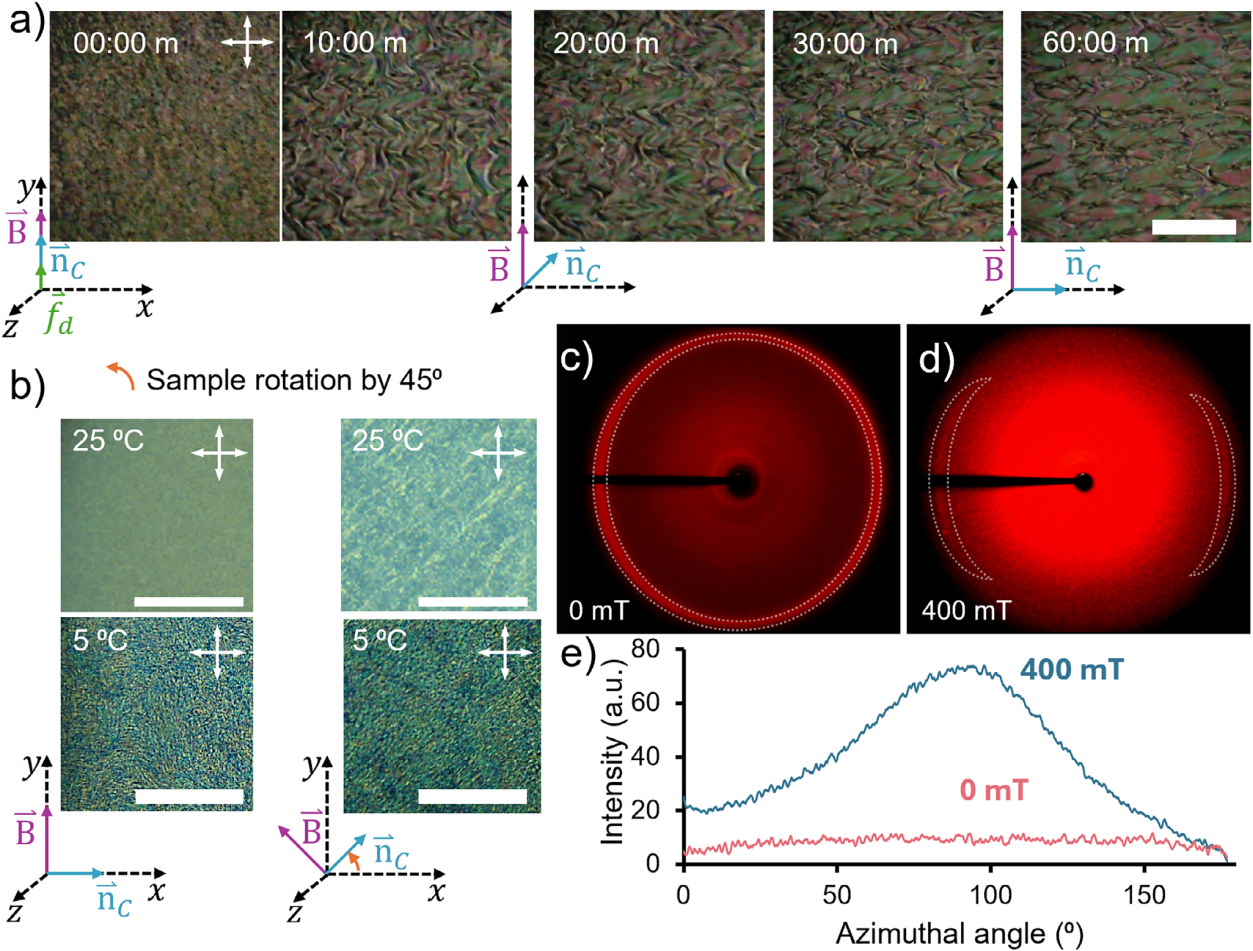
(a) Time‐lapse images of the alignment process in a sample at 5°C under a 400 mT magnetic field. *B* ^⇀^ = magnetic field, 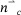 = DSCG director field, 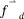 = Capillary flow direction. All vectors are in the xy‐plane. Scale bar: 500 µm. (b) POM images of samples polymerized at 25°C (first row) and 5°C (second row) in the presence of an in‐plane magnetic field of 400 mT. The first column shows the sample aligned to the polarizer, and the second with a 45 ° rotation to highlight the different degrees of alignment. Scale bar: 500 µm. (c,d) are 2D X‐ray diffraction patterns of samples polymerized at 5°C in the presence or absence of an in‐plane magnetic field, respectively. In white dashed lines, the main pattern changes are shown. (e) is the azimuthal angle profile along the outer ring (25.1 < 2θ < 27.5) extracted from (c,d) measurements.

To manipulate the orientation of DSCG aggregates and align their nematic director, LCH precursors were exposed to a strong external magnetic field, expecting that the microstructure of polymerized LCHs could be guided by the deterministic alignment of the LCLC director field within nematic domains. Figure 4a shows the evolution of LCLC alignment and nematic director field over time when the mixtures were exposed to a uniform 400 mT magnetic field using a Halbach array (K = 1). Initially, the mixture was injected by capillary into two hydrophobic glass cells separated by 125 µm plastic spacers. The cell was positioned in the Halbach array in a way that the flow direction was parallel to the magnetic field direction. After cooling the samples to 5°C, finer and randomly shaped nematic domains were formed, which progressed over time into relatively larger and elongated domains perpendicular to the orientation of the magnetic field. DSCG molecules exhibit positive anisotropy of magnetic susceptibility ∆*χ* = 1226 *×* 10*^−^* ^12^ m^3^ */*mol) [14]. When exposed to an external field, they align with their long axis parallel to the field orientation. However, the DSCG molecular stackings forming the nematic phase align orthogonal to the field, defining the nematic director, which is in line with our observations. The evolution of the relatively larger and aligned LCLC domains reached a maximum after 30 min of field exposure and remained stable during the subsequent 30 min conditioning period (Figure 4a).

Although both LCHs prepared at low and ambient temperatures presented a certain degree of birefringence, they showed entirely different textures under POM (Figure 4b). Both LCH samples demonstrated the change in the transmission of polarized light when rotated 45° to crossed polarizers. However, the nematic texture of LCHs prepared at low temperatures was more acute than the nematic texture observed in samples prepared at room‐temperature, which showed faded birefringent marbling. To confirm the transfer of LCLC anisotropy to LCHs after washing DSCG off their network, we conducted X‐ray diffraction (XRD) analysis. Figure 4c,d shows the 2D XRD patterns of LCHs formed at 5°C, in the absence and presence of an external magnetic field, respectively. At 2θ ≈ 26°, we observed a uniform intensity along the circular pattern, confirming the isotropic nature of LCH prepared without a magnetic field (Figure 4c). This contrasted sharply with the formation of two faint and diffuse arcs (Figure 4d) for the LCH fabricated under an external magnetic field. The arcs were oriented parallel to the expected alignment of LCLC domains in LCH networks. This anisotropy can be quantified from the integrated scattering intensity distribution. along the azimuthal angular direction (Figure 4e) by calculating Hermann's order parameter (S) of LCH samples (Note S2), which provides an idea of the molecular degree of ordering in the studied phase. Isotropic samples have *S* ≈ 0, whereas nematic systems usually have an order parameter between *S* ≈ 0.2 − 0.7. The order parameter of a free‐standing LCH prepared in the absence of a magnetic field was S = 0.0, whereas an LCH prepared in the presence of an external magnetic field exhibited S = 0.34. XRD data confirms the anisotropy and higher molecular order within LCH films produced in the presence of a magnetic field.

### 2.3 Bilayers From LCNs and LCHs

**FIGURE 5:**
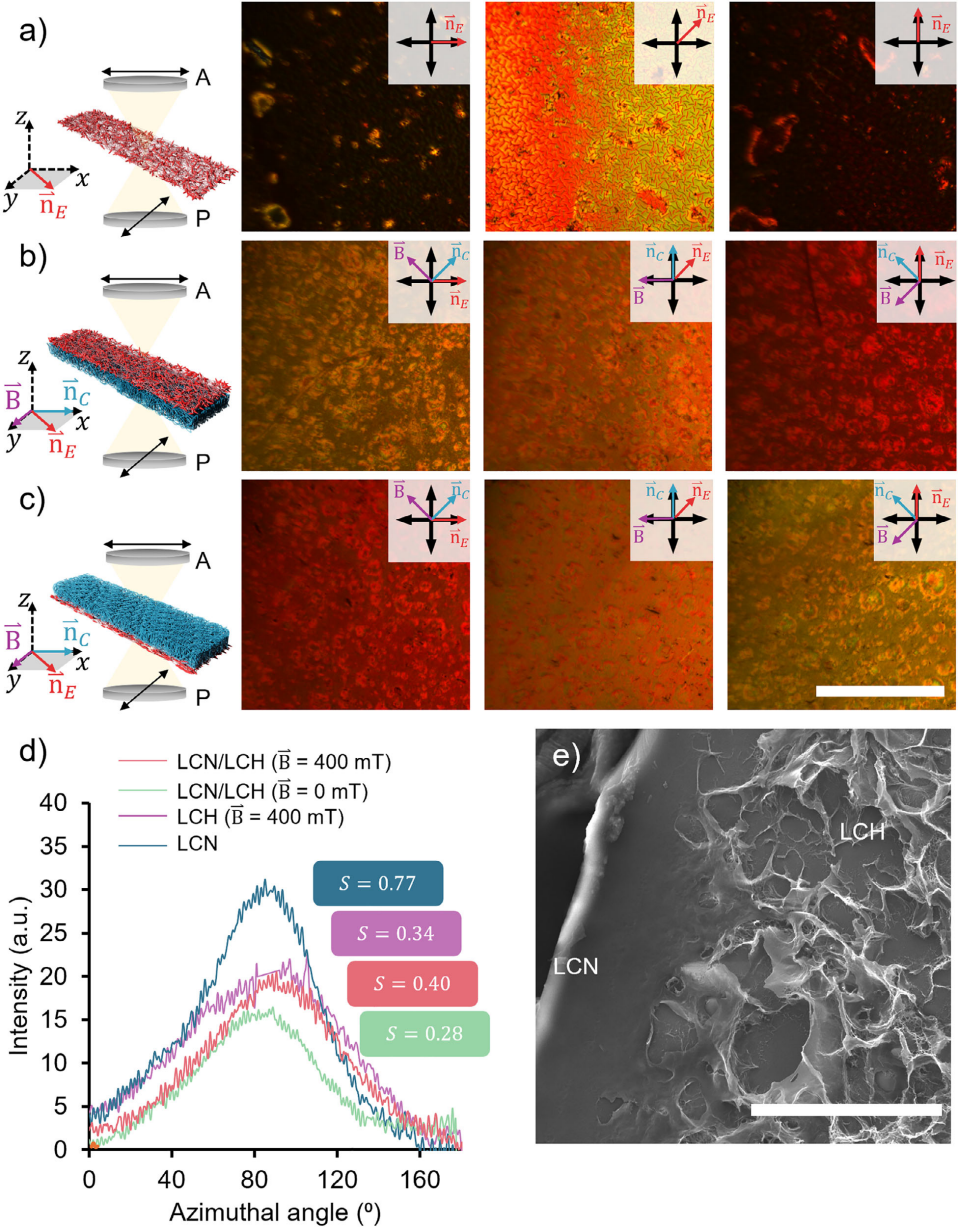
(a) POM images of the LCN film at three different orientations from the analyzer: 0°, 45°, and 90°. (b) POM images of the bilayer composite of LCCLC templated hydrogel and LCN when looking at the LCN side at three different orientations from the analyzer: 0°, 45°, and 90°. (c) POM images of the bilayer composite of LCH and LCN when looking at the LCH side at three different orientations from the analyzer: 0°, 45°, and 90°. In a–c, LCHs were prepared in the presence of a 400 mT magnetic field with a parallel configuration with respect to the LCN director field. The scale bar was 500 µm. (d) Azimuthal angle profile from XRD measurements of the samples produced in a–c. The computed values of the Hermann order parameter, S, are indicated for each series. (e) Representative SEM image of the obtained LCH/LCN composite. Scale bar: 50 µm. 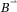 = magnetic field direction. 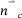 = LCH director field. 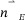
= LCN director field. P = polarizer. A = analyzer.

To obtain LCNs with enhanced porosity and actuation control, we integrated LCHs with LCNs in a bilayer structure. We speculated that the potential interplay between the molecular alignment of LCH and LCN layers might have some impact on the deformation of LCN, and in that scenario, could be used to modulate the shape‐morphing behavior of bilayers. To this end, we prepared LCH layers, with a thickness of 125 µm, between a glass slide and a splay LCN substrate with a thickness of 15–19 µm. Surface modification of the LCN layer with (trimethoxysilyl)propyl methacrylate (TMSPMA) was crucial to promote the adhesion between the two layers. Following LCN surface modification, the DSCG/AAM/PEGDA precursor was injected into a capillary cell with the LCN as the bottom substrate. POM images of a splay LCN monolayer and LCH‐LCN bilayers are shown in Figure 5. The LCN layer exhibited the characteristic texture of a nematic LCN. When rotating the sample, a change in the transmission of light passing through crossed polarizers is observed. Such transmission is minimal when the planar director is parallel to either of the polarizers and is maximum when the director is 45° offset to either of them (Figure 5a). The birefringent nature of LCN‐LCH bilayers was significantly different from that of the pristine LCN. We investigated the impact of LCH anisotropy on the optical properties of the resulting bilayers by magnetically aligning the LCLC templates at a 45° offset relative to the planar director of LCN substrates. POM conducted on either side of the bilayer shows that rotating LCH‐LCN bilayers no longer present a distinct transition between dark and bright (Figure 5b,c), unlike LCN monolayers. Instead, only a gradual increase in intensity was observed when the samples were rotated 45° relative to the polarizer. In the POM images, the director field of the LCN (**n**) is initially parallel to the polarizer before the sample is rotated 45° and 90°. The bilayer sample rotation reveals the contribution of the LCH birefringence, which permits partial light transmission at all angles due to its reduced uniformity and order parameter of the nematic phase, contrasting the highly ordered pristine LCN.

We also used XRD to confirm the overall anisotropy and order within LCH‐LCN bilayers. We tested four samples: a splay LCN monolayer, an anisotropic free‐standing LCH, and two LCH‐LCN bilayers, one with isotropic LCH and the other with anisotropic LCH. The anisotropic LCH bilayer was prepared under a magnetic field parallel to the planar director of the LCN film. Their scattering intensities (integrated at 25.1° < 2θ < 27.5°) vs. azimuthal angle are compared in Figure 5d. The raw scattering intensities (Figure S7) for samples containing LCH were larger than those of LCN, which might be related to the more porous nature and thickness of the samples containing LCHs. Hermann's order parameter calculations for all these samples confirmed that the LCH‐LCN bilayer prepared under an external magnetic field presents a higher degree of molecular order compared to its isotropic LCH‐LCN counterpart. Morphological analysis of LCH‐LCN bilayers by SEM also revealed a marked increase in porosity on the LCH side (Figure 5e), which may enable their application as shape‐morphing drug carrier systems, introducing a novel approach for miniaturized biomedical robotics.

### 2.4 LCH‐LCN Bilayer Actuators in Action

**FIGURE 6:**
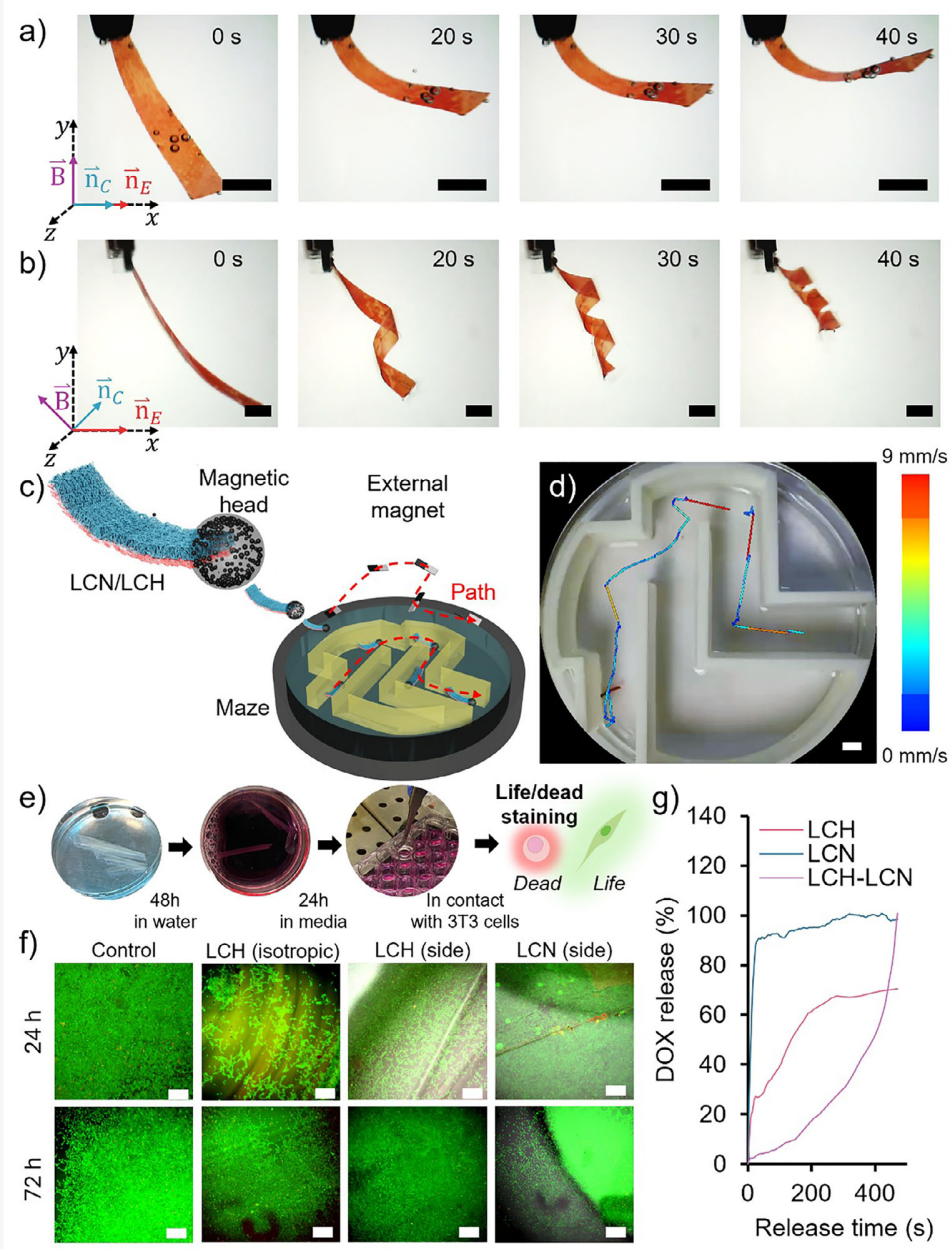
(a,b) are time‐lapse bright field images of LCH‐LCN samples with the LCLC templated director field (⇀nc) aligned in parallel and at 45° to the LCN director field (⇀nE) using external magnetic fields (⇀B) during the polymerization process. Actuation was achieved by increasing the temperature from 15°C to 45°C. Scale bar: 5 mm. (c) Schematic representation of the LCH‐LCN composite integration to a magnetic head composed of UV photopolymerizable glue mixed with MNPs, and its navigation through a complex maze by the application of a constant external magnetic field using a permanent magnet. The dimensions of the robot's tail were 5 mm in width and 25 mm in length. The head was about 5 mm in diameter. (d) Bright field image of the integrated submillimeter robot navigation after tracking its position and local speed. Scale bar: 1 cm. (e) Schematic representation of experimental setup and preparation steps of cell viability studies. (f) Representative live (green) versus dead (red) images of fibroblast viability following cell culture in contact with samples over 24 and 72 h. Scale bar: 400 µm. (g) Profiles of DOX release from samples using UV–vis measurement.

The anisotropic nature of the LCHs prompted us to explore the potential interplay between the anisotropies of the LCN and LCH in bilayers, particularly in regard to their shape‐morphing behavior. Due to the gradient in thermal expansion coefficient in a splay‐aligned LCN, both pristine LCN and multi‐layered cantilevers derived from them undergo out‐of‐plane deformation upon exposure to heat or light. Indeed, minimization of the stored elastic energy arising from the asymmetric order–disorder transition across the two sides of a splay LCN typically results in bending or twisting deformations. Pure bending is observed if their planar director is perfectly parallel to their long axis, and the release of stored elastic energy favors bending. Twisted bending takes place if there is an orientational offset between their planar director and the long axis, and the release of stored energy does not favor pure bending [19]. Within a hybrid LCN cantilever under thermal tension, the stored elastic energy that favors bending (*E_b_*) is proportional to the mechanical and geometrical properties of the layers, as:

$$E_{b}\approx \frac{Yh_{LCN}A}{24\left(1-\nu^{2}\right)}{\left(\frac{h_{LCN}}{h_{Bilayer}}\right)}^{2}$$

where *Y* is the Young modulus, *h_LCN_* is the thickness, *A* is the surface area, and *v* is the Poisson ratio of the LCN cantilever. In our LCH‐LCN design, it is necessary to maintain *h_Bilayer_ >> h_LCN_* in order for the very soft LCH with *Y* in the order of *kPa* to be able to impact or suppress the bending of the LCN with *Y* in the order of *MPa*. We observed that the thickness of our tested LCHs is sufficient to impact and suppress the bending of the underlying LCN layer. difference between the stress distribution across the bilayers with pure bend and Figures 6a and Video S1 show the thermal bending of magnetically aligned LCH‐LCN bilayers inside water, where LCH preferential order is parallel to the planar director of LCN. The bending deformation is significantly reduced compared to that of LCN monolayers (Figure S8). When the preferential order of the LCH is offset by 45° relative to the planar director of the LCN, straight bending is energetically unfavorable, prompting the actuator to twist and adopt a helical configuration (Figure 6b). Photothermal actuation tests conducted underwater revealed a similar trend and behavior (Figure S8 and Video S2). Conducting finite element modeling of the bilayer also showed the onset of twisting for samples with a mismatch between LCH and LCN order (Figure S9). To evaluate the potential use of LCH‐LCN bilayer actuators in remotely controlled small‐scale robotic systems, we equipped them with superparamagnetic iron oxide nanoparticles (MNP), responsive to external magnetic fields. The magnetic modules were made by mixing MNP and optical glue and attaching them to the tip of the bilayer constructs (Figure 6c,d). Thanks to the integrated magnetic module, the bilayer actuator can be precisely navigated along arbitrary trajectories using a permanent magnet. End‐fixed bilayers exhibited pronounced deformation, while free‐standing counterparts displayed modest bending followed by rotational reconfiguration to minimize the stored elastic energy at higher temperatures. Together, these findings establish a compelling proof‐of‐concept for the remote manipulation of LCH‐LCN bilayers, underscoring their promise as shape‐morphing platforms for targeted, minimally invasive drug delivery in dynamic biological environments. While the current navigation demonstrations relied mainly on magnetic pulling, locomotion based on rotating magnetic fields, or self‐propelled motion driven by bilayer cyclic deformation under light, are theoretically viable. However, to implement each of these mechanisms, one needs to optimize bilayers’ mechanical properties, molecular alignment, magnetic particle distribution, and geometry. Future developments in such directions will expand the potential of these hybrid systems toward more realistic untethered soft robotic applications.

### 2.5 LCH‐LCN Bilayer as a Drug Delivery System

To further explore the potential of our bilayer actuator as a drug delivery system, we conducted a series of biocompatibility and drug release studies. When samples were placed in contact with 3T3 cells (Figure 6e; Figure S10), they showed some autofluorescence, albeit stained cells could still be identified. All samples demonstrated high cell viability over the tested timepoints, with very few dead cells (red) observed (Figure 6f). We found that both LCN and LCH samples did not cause cell death, and cells remained alive for at least 72 h. No remarkable differences were found between the cell viability of LCN and LCH samples and that of the LCH‐LCN bilayer.

Having confirmed the cell viability of our materials, we proceeded with further characterization of these samples in terms of drug release. We selected doxorubicin (DOX), a widely used anticancer drug and a standard model in drug delivery research, to evaluate uptake and release behavior. Loading of DOX into samples was carried out by swelling relatively dry samples in solutions of known drug concentration. The loading process revealed a clear influence of the LCH component on the swelling ratio. As shown in Figure S11, the incorporation of an LCH to an LCN in a bilayer led to a substantial increase in swelling ratio, nearly an order of magnitude higher than pristine LCNs. This effect is consistent with the larger hydrophilicity and stronger water absorption of LCH samples compared to LCNs, as demonstrated by contact angle measurements (Figure S12). Such a large swelling ratio of LCH‐LCN samples underscores the potential of LCHs in enhancing drug uptake.

Drug release studies were conducted by immersing drug‐loaded samples in deionized water. Drug release trajectory was monitored under a fluorescent microscope, and kinetics quantified by UV–vis spectroscopy (Figure 6g; Figure S13). A pronounced initial burst of drug release was observed for both drug‐loaded LCN and LCH samples, followed by a gradual release. LCH‐LCN bilayers, however, did not show a burst release, and instead, a sustained, controlled release profile was maintained. Such a large discrepancy between the drug release profiles of LCN, LCH, and LCH‐LCN samples can be attributed to the difference in their porosity and permeability. LCH samples are highly porous and permeable to water, hence absorbing larger amounts of drug and releasing them very fast. LCNs are not permeable to water, and the drug is only absorbed on their surface, leading to a fast release. LCH‐LCN samples, however, benefit from the high porosity and permeability of the LCH layer, while enjoying the controlled and sustained release thanks to the trivial permeability of the LCN layer. Taken together, the results demonstrate that the bilayer actuator combines biocompatibility with tunable drug delivery properties, positioning it as a promising candidate for biomedical applications.

## 3 Conclusion

In this work, we developed hybrid soft actuators from LCNs and LCHs and explored their potential use in small‐scale soft robotics. While LCNs efficiently function as shape‐morphing soft actuators in response to heat, they present limited biocompatibility and porosity. In our design, we take advantage of LCLC templating as a robust method to generate LCHs with tailored morphology and high porosity, as well as potentially higher biocompatibility and drug delivery capability. We demonstrated how the positive anisotropy of magnetic susceptibility of DSCG molecules can be leveraged to control the molecular alignment of their nematic LCLC domains. Magnetic alignment of LCLCs can then be used to program the anisotropic mechanical and optical properties of LCHs devoid of DSCG. The successful integration of LCNs to LCHs offers several advantages. It enhances the porosity of LCNs, potentially reducing their cytotoxicity, while also enabling typically passive LCHs to undergo shape‐morphing in response to external stimuli such as heat or light. To our knowledge, the pronounced and deterministic influence of LCHs templated from LCLCs on the deformation profile of LCNs in a bilayer structure is unprecedented and taps into an unexplored territory in biomedical robotic applications. As a future direction in small‐scale robotics, the twisting motion of LCH‐LCN bilayers could be exploited to achieve corkscrew locomotion under the actuation of external rotating magnetic fields [23, 24, 25, 26]. Such designs could enable controlled drug release in addition to providing shape‐change‐driven locomotion under external stimuli. Indeed, related systems have already demonstrated stimulus‐responsive drug delivery through mechanically induced bending [10]. Future research should also focus on assessing the biocompatibility and drug delivery potential of LCH‐LCN systems and their applicability in biomedical systems. Future work will focus on demonstrating the autonomous locomotion capabilities of the LCH–LCN bilayer actuators. While the current navigation demonstrations relied mainly on magnetic attraction, ongoing efforts aim to achieve self‐propelled motion driven by the bilayer's intrinsic deformation under light or thermal stimuli. Optimizing molecular alignment, magnetic particle distribution, and bilayer geometry will be the next steps that are going to help enable controlled directional movement and more complex navigation behaviors in underwater environments. This development will expand the potential of these hybrid systems toward untethered soft robotic applications.

## 4 Methods

### 4.1 Materials

Mili‐Q‐water, Disodium cromoglycate (DSCG, Sigma‐Aldrich), acrylamide (AA, Sigma–Aldrich), polyethylene glycol diacrylate (TCI America, PEGDA, *n* = 9 approx.), lithium phenyl 2,4,6 trimethyl (LAP, Sigma–Aldrich), brilliant yellow (TCI Chemicals), dimethylformamide (DMF, Sigma–Aldrich), IrgaCure 651 (Fischer Scientific), 4‐((6‐(acryloyloxy)hexyl)oxy)phenyl 4‐(hexyloxy)benzoate (STO3457, Cymit Quimica), 1,4‐Bis[4‐(6‐acryloyloxyhexyloxy)benzoyloxy]‐2‐methylbenzene (RM82, Sigma‐Aldrich), Dispersed Red 1 (DR1, Sigma–Aldrich), dimethyl sulfoxide (DMSO, Sigma–Aldrich), were all purchased from commercial sources. Benzophenone (TCI chemicals), (Trimethoxysilyl)propyl Methacrylate or TMSPMA (stabilized with BHT) (TCI Chemicals), Ethanol (Milipore Sigma), 3T3 Cells (ATCC CRL‐1658), Dulbecco's Modified Eagle Medium DMEM (high glucose, Sigma–Adrich), fetal bovine serum (FBS, Corning), penicillin–streptomycin (PanReac AppliChem), L‐glutamine (Sigma–Aldrich), 24‐well Clear TC‐treated Multiple Well Plates (Corning), Ethidium homodimer‐1 (EthD‐1, ThermoFisher), Calcein (ThermoFisher).

### 4.2 Photopolymerization System

The system developed in this study was designed to fabricate liquid crystal hydrogels, allowing controlled mesogen orientation through a combination of magnetic field alignment and gel solution photopolymerization (Figure S14). The system comprised several key components: a light source, collimator lenses, a sample holder, a Halbach array, and a gear system to control the magnetic field orientation. All components were modeled in Fusion 360 (AutoCAD) and 3D‐printed in PLA using a Prusa i3 MK3S+ printer. The printed parts were assembled onto an MB2025M aluminum breadboard (Thorlabs) using M6 screws. A DC 5–30 V CNC Stepper Motor Controller (PEMENOL) powered a stepper motor (Nema, 17HS10‐0704S) via a driver board (COVVY, TB6600). The illumination system included a liquid light waveguide connected to a collimator lens (SM2F32‐A, Thorlabs) positioned above the setup. For the observation of samples, a white light source was placed beneath the stage. Instead of positioning the collimator lens at the top, a Dino camera with an integrated polarization lens (AM73915MZT, Dino‐Lite) was used.

A uniform planar magnetic field was applied to align the nematic molecules within the solution matrix. This field was generated using a Halbach cylindrical configuration consisting of eight identical N52‐grade NdFeB cubic magnets (25.4 mm, K&J Magnetics) housed in a custom‐designed holder [16]. The holder, designed in AutoCAD Fusion 360 and 3D‐printed from PLA, using a Barcelona 3D Sigma R19 printer, ensured precise magnet positioning. The maximum measured magnetic field strength was 400 mT, concentrated at the device's center. At this position, the polymerizable solution containing magnetic entities was aligned and then polymerized under UV light from an xCite source, projected through the optical system.

To meet the low‐temperature requirements for LCLC templated hydrogel synthesis, we designed and fabricated a cooling holder. This holder consisted of a 25 *×* 25 *×* 25 mm^3^ copper cube with internal cooling channels, designed to maintain a minimum temperature of 5°C by circulating cold water from an external bath. Four 2 mm holes were manually drilled into two opposite faces of the cube to create internal fluid channels, positioned 5 mm from the top surface where the sample was placed. Additionally, two 6 mm holes were drilled to a depth of 10 mm to function as the system's inlet and outlet. Inside each, a further 2 mm hole was drilled to a depth of 22.5 mm, connecting the inlet and outlet to the internal channels.

The initially drilled holes were sealed to prevent leakage, ensuring proper water flow through the cube for effective sample cooling. Cold water from an ice‐water bath was circulated using a peristaltic pump (12 V DC, Kamoer) connected via PC4‐M6 fittings and 4 mm polytetrafluoroethylene (PTFE) tubing.

### 4.3 Sample Preparation

#### 4.3.1 DSCG and Acrylamide Gel Preparation

Programmable hydrogel films were prepared using common lyotropic chromonic liquid crystals, such as DSCG. Briefly, a 0.18 mg mL*^−^* ^1^ aqueous solution of DSCG was mixed with acrylamide (AA) and polyethylene glycol diacrylate (PEGDA) at varying concentrations. To initiate radical polymerization, lithium phenyl‐2,4,6‐trimethylbenzoylphosphinate (LAP) was added at a concentration of 0.002 mg mL*^−^* ^1^ just before polymerization. The samples were polymerized using a photopolymerization setup equipped with a cooling system and a Halbach magnet. Before polymerization, the liquid mixture precursor was introduced into a cell made of two clean glass slides (2.5 × 2.5 cm), which were separated by a 125 µm plastic spacer glued in the cell with optical glue on the sides. The precursor was spread gently through the cell by capillarity to avoid bubbles. Afterward, the cell is placed in the cooling system with a copper block inside the Halbach array to reach the nematic phase of the precursor by lowering the temperature by 5°C, and then polymerized using an ultraviolet light‐waveguide source, coupling with a collimator lens to control the irradiation on the sample.

#### 4.3.2 LCN Synthesis

Prior to preparing the liquid crystal network precursor, capillary glass cells with different LC alignment biases, planar or splay, were fabricated. For glass slides with planar alignment, we used two strategies based on the photoalignment of brilliant yellow (BY) and mechanical rubbing of polyvinyl alcohol (PVA). For the first method, slides were coated with a 1% solution of BY in DMF using a spin‐coating process. The coating was applied in two steps: first, spreading at 600 rpm for 10 s, followed by a second step at 3000 rpm for 60 s. After coating, the slides were placed on a hot plate at 90°C for 30 min, then exposed to polarized blue light (447 nm) for 45 min to induce photoalignment of brilliant yellow molecules, and consequently planar alignment of mesogens deposited on them. In the second method, substrates were spin‐coated with PVA, baked at 90°C–100°C for 30 min, and then mechanically rubbed in one direction using a velvet cloth. Homeotropic substrates were prepared by coating them with polyimide (SE‐5661 Nissan Co.), followed by baking at 90°C–100°C for 30 min. Finally, the two coated slides were assembled with their coated surfaces facing each other, using a 5 µm double‐sided adhesive tape (82600‐2‐18, 3 m (TC)) as a spacer.

The LCN precursor consists of a mixture of diacrylate (M1) and monoacrylate (M2) monomers, a reactive photothermal agent such as Disperse Red 1 (DR1), and Irgacure 651 as the photoinitiator to drive the polymerization reaction upon UV exposure. The synthesis of LCNs in splay alignment involves the capillary‐driven spreading of the LCN precursor in the isotropic phase (100°C–105°C), into a cell formed by two glass substrates with homeotropic and planar alignment coatings. The cell was slowly cooled (at 1°C/min) until it reached the nematic phase (at 45°C–55°C). These substrates are critical for inducing the splay alignment responsible for bending deformation under photothermal stimuli [27]. This splay geometry (planar‐homeotropic) creates a gradient of director orientation through the film thickness, generating differential strain upon heating and thereby promoting bending deformation, as previously reported for splayed LCN/LCG constructs that exhibit bending with heating [27]. The precursor is then polymerized under UV light (365 nm) for 30 min to achieve complete crosslinking. Polymerization was triggered using a custom‐built light‐waveguide coupling system adapted for microscopy applications.

#### 4.3.3 Chemical Modification of the LCN Surface and LCH Integration

The objective of modifying the surface on the LCN film is to change the properties of the side in contact with the hydrogel layer to have new functional groups to enhance the adhesion and facilitate subsequent reactions or the adhesion of the hydrogel layer, creating a strong bond between layers and avoiding delamination.

The process starts once the synthesis of the liquid crystal elastomer in splay or planar alignments is completed. First, the samples need to be exposed for about 10–15 min in a UV ozone cleaner to increase the surface energy and make the surface more chemically reactive to other processes. Subsequently, the surface of the LCN was coated with a silane. The latter can react with the external surface layer of the LCN, resulting in a chemically modified surface with new functional groups to enhance adhesion and facilitate subsequent reactions [28]. TMSPMA was used for this process. However, due to the easy reactivity of this compound with the water and oxygen present in the air, controlled environment systems are necessary to avoid secondary reactions that can affect the LCN samples’ modification. The samples are placed on a glass substrate with the expected side to be modified (in the case of splay LCN with a planar side up). Later, the samples were collected in a vial or glass petri dish, and the open side was covered in aluminum foil with small holes for an inlet and outlet. A syringe connected to a nitrogen gas source was placed in the inlet hole with a low nitrogen flow, in order to circulate nitrogen in the system, thus creating an inert environment. After 5 min of nitrogen purging, TMSPMA was injected into the system until the samples were completely covered in silane. We allowed 30 min of reaction time to achieve proper bonding.

TMSPMA is a common adhesion promoter that has two distinct reactive ends. The end terminated by methoxy, (−Si(OCH3)3), anchors to the surface of the LCN substrate through a hydrolysis reaction. With ambient humidity, relatively inert methoxy groups convert into highly reactive silanol groups (−Si(OH)3), which are primary actors in forming strong, stable bonds with the substrate surface. The methacrylated end of the TMSPMA is structurally analogous to the AAM and PEGDA monomers used in the formation of our hydrogels. The double bond (C═C) undergoes free‐radical photopolymerization, becoming a covalently integrated component of the resulting hydrogel network [28]. Note that the interfacial adhesion between the hydrogel and LCN is highly dependent on the yield of the reaction between activated LCN and TMSPMA and was challenging to control through our experimental capabilities. Our bonding success rate was not always perfect. To improve the bonding and reproducibility of surface modification, a systematic study is needed, and perhaps the utilization of chloro or thiol‐terminated silane molecules that traditionally have higher activity. This is outside of the scope of our studies.

To fabricate hybrid structures of LCN and LCH, the LCN sample was placed between two clean glass slides, separated by a 125 µm‐thick plastic film (Artus 125, from Artus Corp., USA). The LCH precursor was then introduced into the cell and polymerized at either room‐temperature or under cooling conditions on top of the fabricated LCN film.

#### 4.3.4 Magnetic Nanoparticles: Synthesis and Integration Into the Composite

In an alternative approach, and inspired bythe literature, magnetic particles (Fe_3_O_4_) were dispersed into the LCN precursor prior to polymerization and bilayer fabrication [24]. The dispersion process involved adding the Fe_3_O_4_ particles to the precursor along with a small amount of chloroform to aid in uniform particle dispersion. The resulting mixture was then heated to evaporate the chloroform. Once the chloroform was fully removed, the precursor containing the magnetic particles was introduced into the splay alignment cell as described previously. The sample was then quenched from the isotropic phase (100°C) to the nematic phase (45°C–55°C) to increase the matrix viscosity and suppress nanoparticle aggregation. Polymerization was subsequently performed under UV light (365 nm) for 30 min to crosslink the LCN and fix the magnetic particles within the network. This approach results in an LCN film with integrated magnetic particles, enabling potential magnetically responsive functionalities. Magnetic iron oxide nanoparticles were synthesized using the precipitation method. Briefly, 10 g FeCl_3_ and 4.05 g FeCl_2_ were dissolved in 550 mL of deionized water under magnetic stirring at 90°C. Then, 30 mL of NH_4_OH solution (30 wt.%) was gradually added (10 mL/min) to the stirring iron salt solution to anticipate the redox reaction. The reaction vessel was maintained at 90°C under magnetic stirring for 1 h. The resulting precipitate was collected, followed by rinsing with deionized water and ethanol, and dried in the air. Then, we proceed to the normal procedure to fabricate the bilayer based on the junction of the LCN and LCH.

#### 4.3.5 Cell Culture and Biocompatibility Studies

To perform the corresponding cytotoxicity studies, we performed live‐dead analysis by exposure of 3T3 cells to the different samples. We used NIH/3T3 mouse embryonic fibroblasts and regular complete growth medium for cell culture. DMEM was supplemented with 10% (v/v) fetal bovine serum (FBS), 1% (v/v) penicillin–streptomycin (final concentration: 100 U/mL penicillin and 100 µg/mL streptomycin), and 1% (v/v) L‐glutamine (200 mm). The prepared growth medium was stored at 4°C and used within 4 weeks, and it was warmed to 37°C before cell culture experiments.

In order to prepare the 3T3 cells for cytotoxicity studies, 200 µL of media with a cell concentration of 5 × 104 cells/mL was added to each well of a 24‐well plate, leaving them to attach overnight. In parallel, the different materials under study underwent several washing steps (48 h in water and 24 h in media) in order to release any non‐polymerized monomer. After washing, small sections of the samples were added to each well, where a PDMS ring was used to ensure full contact of the materials with the 3T3 cells during the biocompatibility studies. Two different sets of samples, one for 24 h and another for 72 h viability studies, were prepared, with three replicates for each sample.

Cell viability was assessed by staining with calcein, which labels metabolically active (live) cells with green fluorescence, and a red fluorescent nuclear dye (EthD‐1) selective for membrane‐compromised (dead) cells. After the required incubation time between the samples and the cells (24 and 72 h, respectively), the media was removed. A volume of 200 µl of calcein at 2 µM and EthD‐1 at 4 µm was added to each well, keeping them in the incubator for 30 min at 37°C and 5% CO2. Following incubation, all samples were examined using a fluorescence microscope, and images were analyzed using Fiji software [29].

#### 4.3.6 Drug Iptake and Release

To monitor the drug release, UV–vis measurements were performed by placing the DOX‐loaded samples at the bottom of a cuvette, followed by the addition of 1 mL of deionized water. Measurements were started immediately after adding the water, with illumination of cuvettes to a scanned light ranging from 450 to 550 nm at a rate of 100 nm/min for 100 cycles. The concentration of released DOX in the water was calculated using the Beer–Lambert law $\left(c=\frac{A}{\varepsilon l}\;\right)$, where *c* is the concentration of DOX, *A* is the absorbance at 485 nm (the peak absorption wavelength for DOX), ε is the molar absorptivity of DOX obtained from the slope of the absorbance–concentration calibration curve at 485 nm, and l is the light path length of the cuvette. The DOX concentration over time was converted to the mass of DOX released and normalized by the initial DOX content in the sample prior to release. Samples of hydrogel and LCN were swollen in water, and their surfaces were gently dried using Kimwipes. A droplet of water was then placed on the surface of each sample to assess its hydrophilicity.

A piece of a hydrogel sample fully swollen in DOX solution was dried overnight. The sample was then placed under a fluorescence microscope (Nikon Eclipse Ti2) and exposed to an excitation wavelength of 475 nm, corresponding to the DOX excitation peak. A droplet of water was subsequently added to the sample to visualize the DOX release process.

#### 4.3.7 Swelling Experiments

Samples of hydrogel, LCN, and hydrogel–LCN bilayer were left to dry overnight at room‐temperature. The masses of the samples were recorded before swelling. The samples were then swollen in a 5 µg/mL doxorubicin (DOX) aqueous solution overnight. After gently wiping the surface of the samples with Kimwipes, their masses were recorded as swollen. The samples were subsequently dried overnight once more, and the masses were recorded after drying. The mass swelling ratio was calculated as the mass of absorbed DOX solution normalized to the initial mass of the sample before swelling, according to the following equation: $\frac{M_{swolle}-M_{init}}{M_{init}}$.

### 4.4 Materials Characterization

Rheological analyses were performed using a TA Discovery H30 rheometer fitted with a UV curing accessory and an upper Peltier plate. Parallel plates were utilized for the measurements. Time sweep tests were conducted at a frequency of 1.6 Hz and a strain of 1%. UV exposure at an intensity of 10 mW*/*cm^2^ was initiated 30 s after data collection began and sustained for the entire duration of the experiment (300 s). Flow sweep tests were carried out with shear rates ranging from 0.001 to 1000 s*^−^* ^1^. All experiments were conducted at 5°C and 25°C.

Two‐dimensional X‐ray diffraction (2D‐XRD) patterns were recorded in transmission mode with a Bruker Venture DUO equipped with a Photon III detector using a Cu Kα X‐ray Micro‐source beam with a wavelength (λ) of 0.154184 nm at 1.10 mA, 50.00 kV, a frame exposure of 300 s. The detector was centered at 2*theta = 0 with a 90 mm sample to the detector distance. 1D azimuthal integrated intensity distributions of the 2D plots were calculated post‐testing with the instrument software. Data processing was performed in OriginPro, where (for the sake of plotting) the data were phase shifted, denoised, baseline corrected, and then normalized.

For scanning electron microscopy (SEM) analysis, hydrogel samples were frozen in liquid nitrogen and subsequently freeze‐dried for 24 h using a Labconco system. The dried samples were coated with a 5 nm gold layer following standard procedures before being examined using an FEI Quanta FEG 250 environmental scanning electron microscope (ESEM) equipped with energy‐dispersive X‐ray spectroscopy (EDX). Cross‐polarized and bright‐field images were obtained using a Euromex iScope polarized microscope. A temperature‐controlled heating stage (Linkam, TMS93/LNP94) was employed to regulate sample temperature during imaging. Image characterization was performed by using Fiji (Image J) image treatment software, evaluating the pore size using the “Particle Analysis” plug‐in.

Differential scanning calorimetry (DSC) experiments were performed using a TA Instruments Q2000 system equipped with an RCS90 cooling unit. A 20 mg sample was sealed in a hermetic pan, and a heating protocol was applied. The sample was initially cooled to 5°C before being heated to 80°C at a rate of 5°C per minute. This cycle was repeated twice for each sample.

In order to understand the difference in porosity between our studied samples, we ran BET measurements. Approximately 0.1 g of the powdered sample was weighed into a clean glass sample tube. The sample was then degassed under vacuum at 80°C for 15 h using a Micromeritics VacPrep 061. The sample was then analyzed using a Micromeritics Gemini VII 2390a Surface Area Analyzer with nitrogen (N_2_) as the adsorbate at liquid nitrogen temperature. BET surface area was measured using the relative pressure range p/p° = 0.05–0.30, while total pore volume was determined at p/p° = 0.99.

This project was financially supported by Natural Sciences and Engineering Research Council of Canada (NSERC), the European Union's Horizon 2020 Proactive Open program under grant agreement No 952152 (Magnetically steerable wireless Nanodevices for the tarGeted delivery of therapeutIc agents in any vascular rEgion of the body (ANGIE)) the Ministerio de Ciencia, Innovación y Universidades (grant PID2020‐116612RB‐C33 funded by MCIN/AEI/10.13039/501100011033), and the Generalitat de Catalunya (2021 SGR 00270). I.H.T.G. acknowledges the financial support from the National Secretariat of Science, Technology, and Innovation of Panama (SENACYT) for funding his Master's studies. S.P. and J.P.‐L. also acknowledge support from the European Union's Horizon Europe Research and Innovation Programme under the EVA project (GA no: 101047081) and the Swiss State Secretariat for Education, Research and Innovation (SERI). J.P.‐L. and M.G. acknowledge the Agencia Estatal de Investigación (AEI) for the María de Maeztu project no. CEX2021‐001202‐M. J.P.‐L. acknowledges financial support from Departament de Recerca i Universitats: del Departament d'Acció Climàtica, Alimentació i Agenda Rural; i del Fons Climàtic de la Generalitat de Catalunya (2023 CLIMA 00011). J.I‐M. Acknowledges financial support from MICIU/AEI/10.13039/501100011033 (Grant No. PID2022‐137713NB‐C21). O. B. acknowledges a Joan Oró FI fellowship (2023 FI‐3 00065) by the Secretariat of Universities and Research of the Department of Research and Universities of the Generalitat of Catalonia and the European Social Plus Fund. J.T‐A. acknowledges financial support from the funding of a FPU fellowship FPU22/01916 from  MICIU. C. W. V. J. and M. G. acknowledge the financial support from the Spanish Ministry of Science through the grant PID2023‐151682NA‐I00, as well as from the Agencia Estatal de Investigación (AEI) Unidades de Excelencia María de Maeztu 2021 CEX2021‐ 001202‐M. M. G. also acknowledges the Spanish Ministry of Science through Ramon y Cajal Grant No. RYC2020‐945030119‐I and the financial support from MCIN/AEI/10.13039/501100011033/ and FEDER (Grant No. PID2023‐151682NA‐I00). H.S. acknowledges the assistance of Dr. F. Salim and C. dal Castel from Analytical Labs of the Department of Chemical Engineering at the University of Waterloo in BET analysis.

## Conflicts of Interest

The authors declare no conflict of interest.

## Supporting information




**Supporting File 1**: adma72500‐sup‐0001‐SuppMat.docx.

**Supporting File 2**: adma72500‐sup‐0002‐VideoS1.mp4.

**Supporting File 3**: adma72500‐sup‐0003‐VideoS2.mp4.

## Data Availability

The data that support the findings of this study are available in the supplementary material of this article.
