# Supplementary material for: Porous Hybrid Soft Actuators From Liquid Crystal Networks and Lyotropic Chromonic Liquid Crystal Templated Hydrogels
Source: Adv Mater. 2026 Feb 15;38(24):e16677. doi: 10.1002/adma.202516677 (PMC13113223; doi:10.1002/adma.202516677)
Supplement: Supplementary file 1 — Supporting File 1: adma72500‐sup‐0001‐SuppMat.docx. [file ADMA-38-e16677-s001.docx]

Supporting Information

Porous Hybrid Soft Actuators from Liquid Crystal Networks and Lyotropic Chromonic Liquid Crystal Templated Hydrogels

*Ramón Santiago Herrera Restrepo^#^, Irving Hafed Tejedor García^#^, Matthew Gene Scarfo, Negin Bouzari, Negar Rajabi, Olga Bantysh, Joel Torres-Andrés, Christopher W. V. James, Maria Guix, Amirreza Aghakhani, Jordi Ignés-Mullol, Salvador Pané, Josep Puigmartí Luis^*^, and Hamed Shahsavan ^*^*

Dr. R. S. Herrera Restrepo, Dr. C. James, Dr. M. Guix, Prof. Dr. J. Puigmartí-Luis, Departament de Ciència de Materials i Química Física, Institut de Química Teòrica i Computacional, Universitat de Barcelona, Barcelona 08028, Spain & Catalan Institution for Research and Advanced Studies (ICREA), Passeig de Lluís Companys, 23, Barcelona, 08010, Spain

O. Bantysh, J. Torres-Andrés, Prof. Dr. J. Ignés-Mullol Departament de Ciència de Materials i Química Física, Institute of Nanoscience and Nanotechnology (IN2UB), Universitat de Barcelona, Barcelona 08028, Spain

Prof. Dr. A. Aghakhani, Institute of Biomaterials and Biomolecular Systems (IBBS), University of Stuttgart, Pfaffenwaldring 57, Stuttgart 70569, Germany

Prof. Dr. Salvador Pané, Multi-Scale Robotics Lab, Institute of Robotics and Intelligent Systems, ETH Zurich, Tannenstrasse 3, Zurich 8092, Switzerland

I. H. Tejedor, M. G. Scarfo, N. Bouzari, N. Rajabi, Department of Chemical Engineering, University of Waterloo, 200 University Ave. W, Waterloo, ON N2L 3G1, Canada

Prof. Dr. H. Shahsavan, Department of Chemical Engineering, Waterloo Institute for Nanotechnology, Institute for Polymer Research, and Center for Bioengineering and Biotechnology, University of Waterloo, 200 University Ave. W, Waterloo, ON, N2L 3G1, Canada

# Authors with equal contribution

*Corresponding author: [josep.puigmarti@ub.edu](mailto:josep.puigmarti@ub.edu), [hshahsav@uwaterloo.ca](mailto:hshahsav@uwaterloo.ca)

**Table of Contents**:

**Supplementary Note 1**: Representative polarized optical images (POM) of precursor with different concentrations of Monomer and Crosslinker

**Supplementary Figure 1**: Representative polarized optical images (POM) of precursor with different concentrations of Monomer and Crosslinker

**Supplementary Figure 2**: Scanning differential calorimetry analysis of the formulations containing disodium cromoglicate (DSCG) at 18 %, and acrylamide (AAM) and polyethylenglycol diacrylate (PEGDA) at different concentrations.

**Supplementary Note 2**: Gelation process in terms of cross-over time and tan δ.

**Supplementary Note 3**: Rheological Characterization of Precursor Solutions via Flow and Amplitude Sweep Measurements

**Supplementary Figure 3**: Rheological Characterization of Precursor Solutions via Flow and Amplitude Sweep Measurements

**Supplementary Figure 4**: Rate and slope evaluation of the photogeology processes at a) 25 ºC and b) 5 ºC.

**Supplementary Figure 5**: Evaluation of the storage (G′) and loss (G″) moduli ratio (tan δ) during the polymerization process.

**Supplementary Figure 6**: BET single point surface area.

**Supplementary Note 4**: Calculation of Hermann’s Order Parameter

**Supplementary Figure 7**: Raw XRD^2^ data, phase corrected.

**Supplementary Figure 8**: Deformation of LCN and LCH-LCN bilayers

**Supplementary Figure 9**: FEM Modelling of bilayer actuators

**Supplementary Figure 10**: Time-lapse images of the actuation process of the robot with 45 º misalignments between the director field of the liquid crystal elastomer and the lyotropic chromonic liquid crystal templated acrylamide/polyethyleneglycol diacrylate gel.

**Supplementary Figure 11**: Swelling process and drug loading.

**Supplementary Note 5**: Swelling data

**Supplementary Figure 12**: a) Representative images of the swelling and drug loading process of the LCN, LCH and bilayer LCH-LCN. b) Mass change ratio of the results obtained in a) for each of the studied samples.

**Supplementary Figure 13**: Images of the contact angle test on a LCH substrate (a) and on a LCN substrate (b).

**Supplementary Figure 14**: a) Sequence example of fluorescence microscopy images of DOX release. b) Normalized intensity measurement of the locations indicated in a) as SQ1 and SQ2 for DOX release.

**Supplementary Figure 15**: Schematic representation of the photopolymerization system and the cooling system for producing aligned LCLC templated hydrogels

**Supplementary Note 1: Representative polarized optical images (POM) of precursor with different concentrations of Monomer and Crosslinker**

POM images of hydrogel precursor formulations with varying concentrations of acrylamide monomer and PEGDA crosslinker: This figure was used to evaluate the effect of precursor composition on the homogeneity and distribution of phase-separated domains. The goal was to identify the formulation that yields the most uniform distribution and optimal morphology for hydrogel synthesis. Notably, increasing PEGDA and AAM concentrations tends to enhance phase separation.


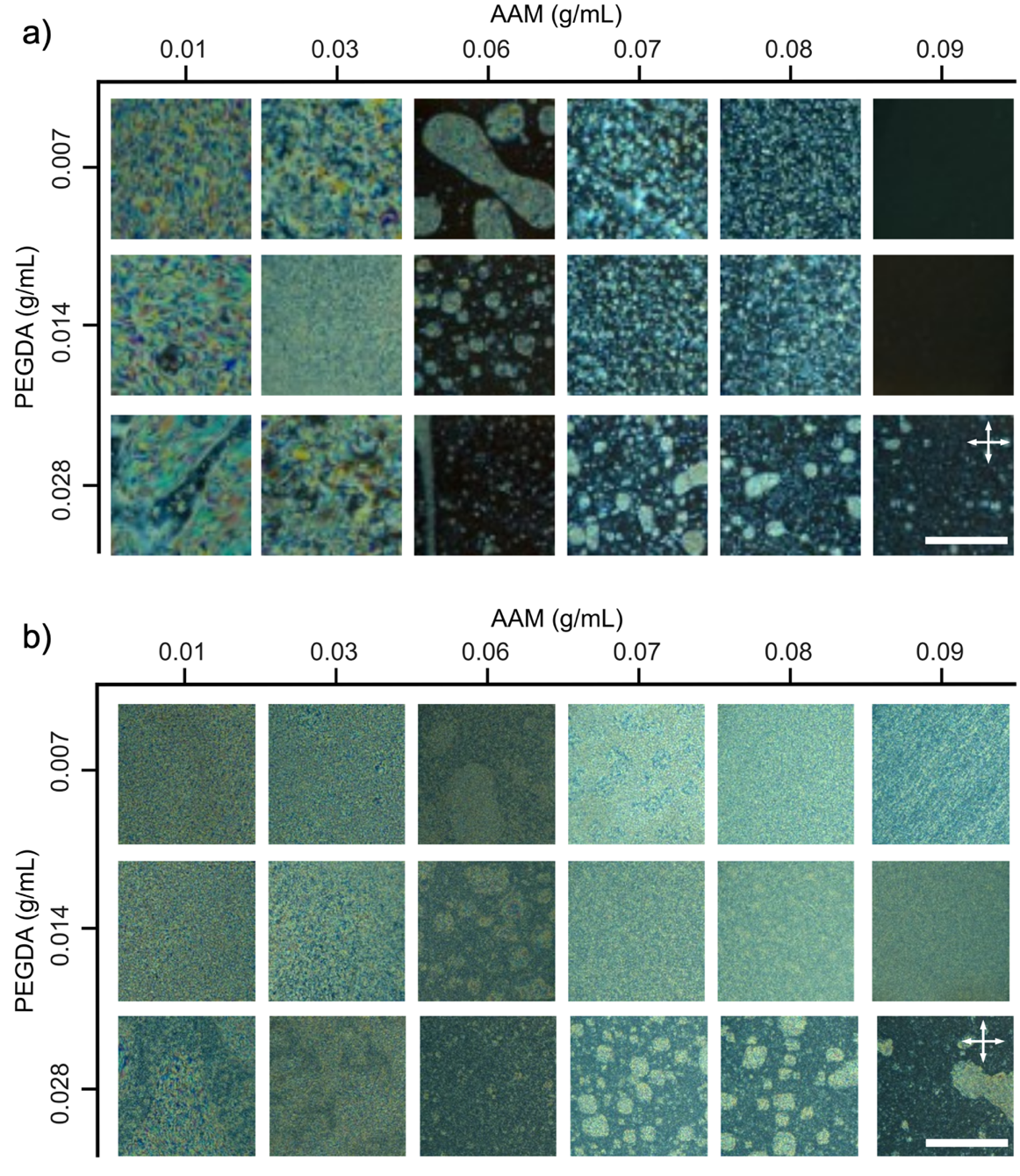


**Figure S1.** a) Representative polarized optical images (POM) of solutions consisting of disodium cromoglicate at 0.18 mg/mL, and acrylamide (AAM) and polyethylenglycol diacrylate (PEGDA) at varying concentrations. b) Representative POM of photopolymerized solutions produced in a) after addition of lithium phenyl-2,4,6-trimethylbenzoylphosphinate (LAP) at 0.002 g/mL. Scale bar: 500 μm


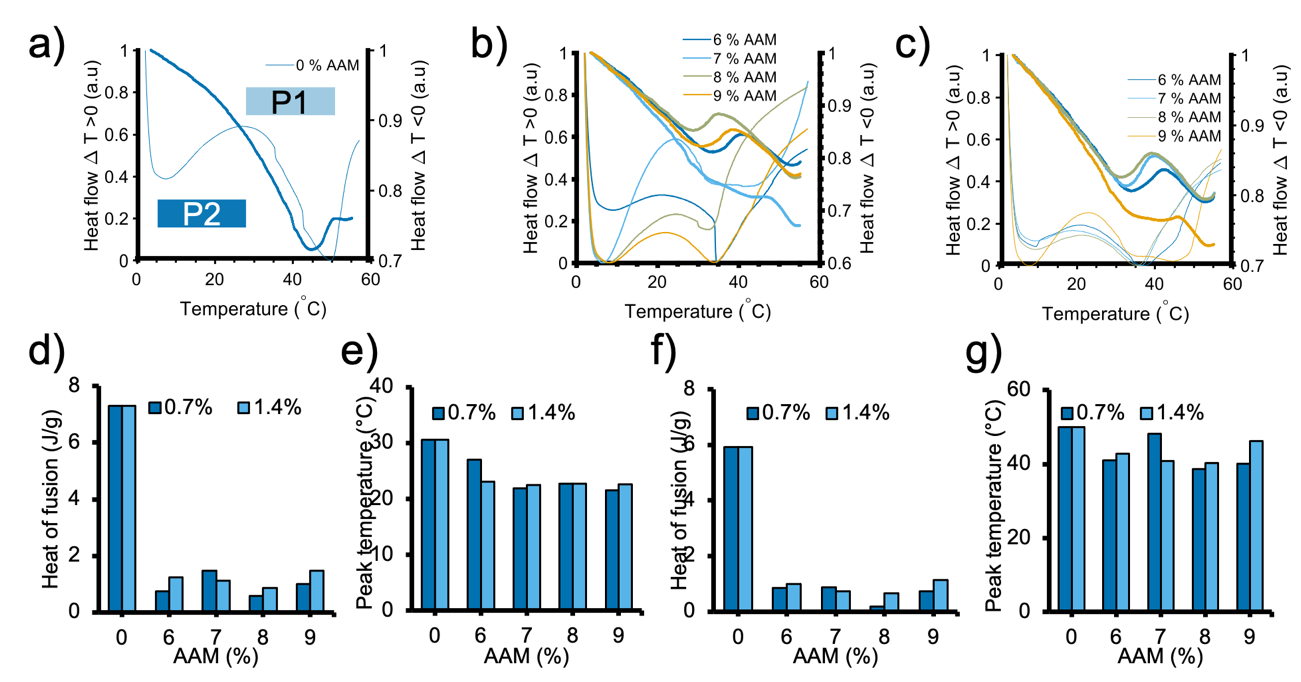


**Figure S2.** Differential Scanning Calorimetry analysis of the formulations containing disodium cromoglicate (DSCG) at 18 %, and acrylamide (AAM) and polyethylene glycol diacrylate (PEGDA) at different concentrations. a) SDC measurement of a solution consisting of only DSCG at 0.18 mg/mL. P1 and P2 refer to measurements where the temperature was increased from 0 ºC to 60 ºC, and decreased from 60 ºC to 0 ºC, respectively. b) Effect of adding AAM to a solution containing DSCG at 18 % and 0.7 % of PEGDA. c) Effect of adding AAM to a solution containing DSCG at 18 % and 1.4 % of PEGDA. In b) and c), the thick and thin curves correspond to experiments where the temperature was increased from 0 ºC to 60 ºC, and decreased from 60 ºC to 0 ºC, respectively. d) and e) correspond to the Heat of fusion (area below the found peak) and the peak temperature for the increased temperature experiments in solutions of DSCG at constant 18 %, and varying AAM and PEGDA concentrations. f) and g) correspond to the Heat of fusion (area below the founded peak) and the peak temperature for the decreased temperature experiments in solutions of DSCG at a constant 18 %, and varying AAM and PEGDA concentrations.

**Supplementary Note 2: Rheological Characterization of Precursor Solutions via Flow and Amplitude Sweep Measurements**

In the flow sweep test (figure S3-a), the data show a significant decrease in viscosity with increasing shear rate, indicating shear-thinning behavior, especially at 5 °C. Amplitude sweep measurements (figure S3-b) were performed at a constant temperature (5 °C) and frequency (1 Hz) to evaluate the complex viscosity (Pa·s) as a function of oscillatory strain (%). At low strains (<0.1%), the complex viscosity remains relatively stable, followed by a notable decrease as strain increases, indicating the onset of the nonlinear viscoelastic region. These results confirm that the precursor exhibits suitable rheological characteristics for controlled processing. The high viscosity at low temperatures can aid in alignment of chromonic domains (e.g., homeotropic alignment in cells) and maintain spatial uniformity during photopolymerization.


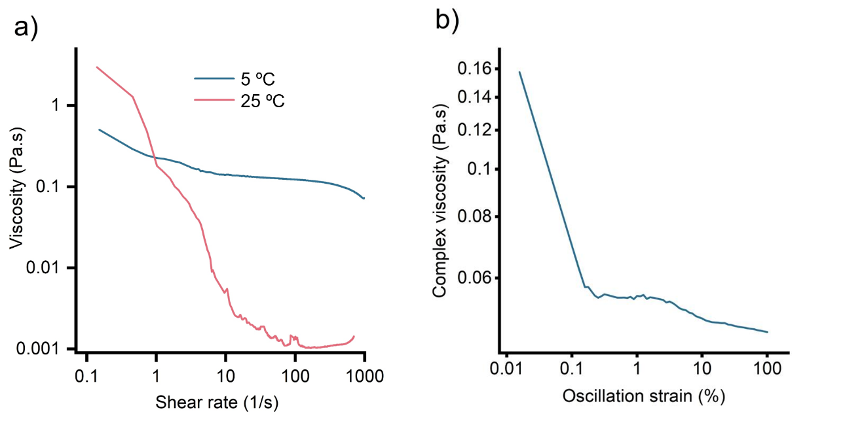


**Figure S3**. **Rheological Characterization of Precursor Solutions via Flow and Amplitude Sweep Measurements** a) Flow swept measurement of the initial precursor solutions containing DSCG at 0.19 mg/mL, AAM at 0.09 mg/mL, PEGDA at 0.007 mg/mL, AAM at 0.09 mg/mL, PEGDA at 0.007 mg/mL, and Lithium phenyl-2,4,6-trimethylbenzoylphosphinate (LAP) at 0.002 mg/mL at two different temperatures. b) Amplitude swept measurement of the initial precursor at constant temperature (5 °C) and constant frequency (1 Hz), and the complex viscosity (Pa·s) as a function of the oscillation strain (%). At low strain (< 0.1%).


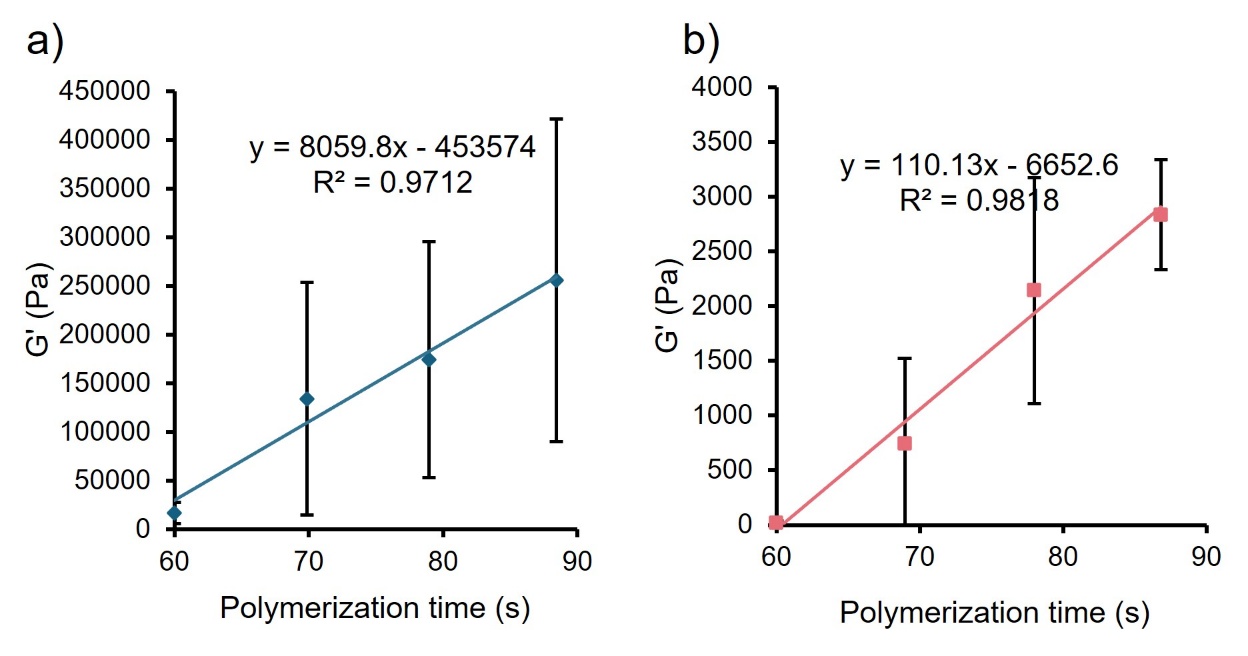


**Figure S4**. Rate and slope evaluation of the photorheology at a) 25 ºC and b) 5 ºC

**Supplementary Note 3: Gelation process in terms of cross-over time and tan δ.**

The evolution of the viscoelastic moduli during photopolymerization revealed that the gelation point, i.e., defined by the crossover of the storage (G′) and loss (G″) moduli, occurred at approximately the same time for both temperatures, with *gelation time* values of 60.5 s at 5 °C and 61.3 s at 25 °C. This similarity indicates that the onset of network formation is primarily controlled by the photoinitiation process rather than by diffusion-limited propagation. However, the magnitude of the moduli at the crossover differed markedly, increasing from 55 Pa at 5 °C to 3.27 × 10⁴ Pa at 25 °C. The substantially higher crossover modulus at 25 °C reflects a more efficient crosslinking process and the formation of a denser, mechanically stronger network at the gel point. In contrast, the much lower modulus observed at 5 °C suggests reduced segmental mobility and hindered radical propagation, yielding a softer and more weakly connected gel. The temporal evolution of *tan δ* during photopolymerization provides further insight into the viscoelastic behavior and network development at different curing temperatures (Figure S5). Before illumination, the system at 5 ºC exhibited *tan δ* > 1, confirming viscous liquid behavior. Because of the limitation of our rheological setup to measure low viscous fluids, experiments performed at 25 ºC showed a *tan δ* ≈ 1, which we interpreted as a system with liquid behavior. Upon light exposure, *tan δ* rapidly decreased, marking the transition from liquid- to solid-dominated response. The gelation point, identified at *tan δ* ≈ 1, occurred at approximately 60 s for both 5 °C and 25 °C, consistent with the crossover times obtained from G′ and G″. After gelation, the *tan δ* values stabilized at markedly different levels depending on temperature: ≈ 0.3 at 25 °C and ≈ 0.1 at 5 °C. The latter different ratios indicate that at 5 °C the cured material became predominantly elastic, reflecting efficient network formation and higher crosslink density. Conversely, the higher *tan δ* retained at 25 °C denotes a more dissipative gel. The differences between these values can be correlated with the fact that the process at 25 ºC involves the transition from an isotropic liquid to a gel, whereas at 5 ºC the transition occurs from the nematic phase. We believed that the presence of a nematic phase changes the kinetics of the reaction, altering the mechanical properties of the final gel, where the gel produced at 5 ºC showed smaller values of G’ and G’’ in contrast to the gel produced at 25 ºC. In general, these trends confirm that, while gelation onset is governed primarily by the photo-initiation step, the post-gelation viscoelastic evolution and the final mechanical character of the network are strongly temperature-dependent.


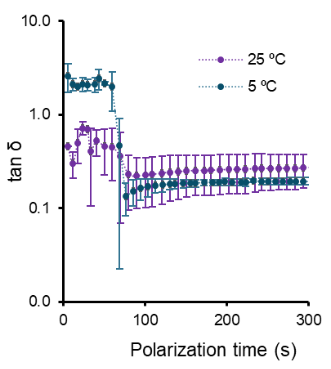


**Figure S5.** Evaluation of the storage (G′) and loss (G″) moduli ratio (tan δ) during the polymerization process.

**Supplementary Note 4: BET Analysis on LCN and LCH samples**

In addition, to highlight the porosity variations introduced by the incorporation of LCH, BET surface area measurements were conducted, as presented in **Figure S6**. The single-point surface area analysis revealed that LCH exhibited a porosity approximately twentyfold greater than that of LCN. Furthermore, its integration within the LCH–LCN bilayer markedly enhanced the porosity of the LCN component.


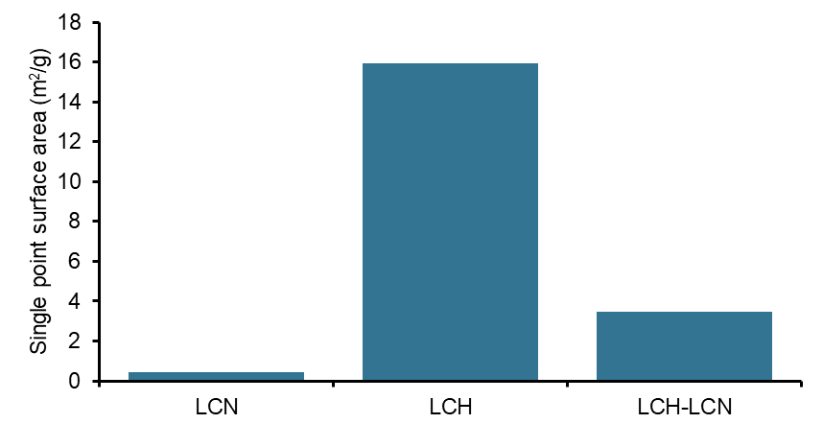


**Figure S6.** BET single point surface area at a relative pressure (p/p°) of 0.30

**Supplementary Note 4: Calculation of Hermann’s Order Parameter**

The order parameter, **S (Equation supplementary 1)**, was calculated by the following equations. **γ** represents the angle between the (200) lattice plane and the alignment direction of the polymer chains **(Equation supplementary 2)**, **Φ** is the azimuthal angle, and I(Φ) is the 1D integrated intensity distribution **(Equation supplementary 3)**.


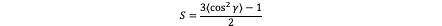
Supplementary Equation 1


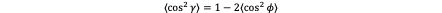
 Supplementary Equation 2


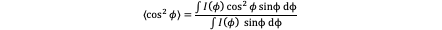
Supplementary Equation 3


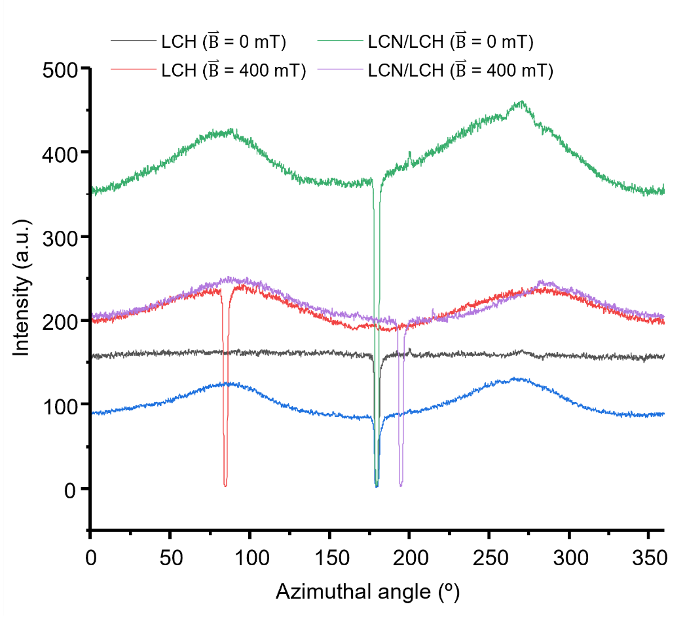


**Figure S7**. Raw XRD^2^. Raw XRD^2^ data, phase corrected.


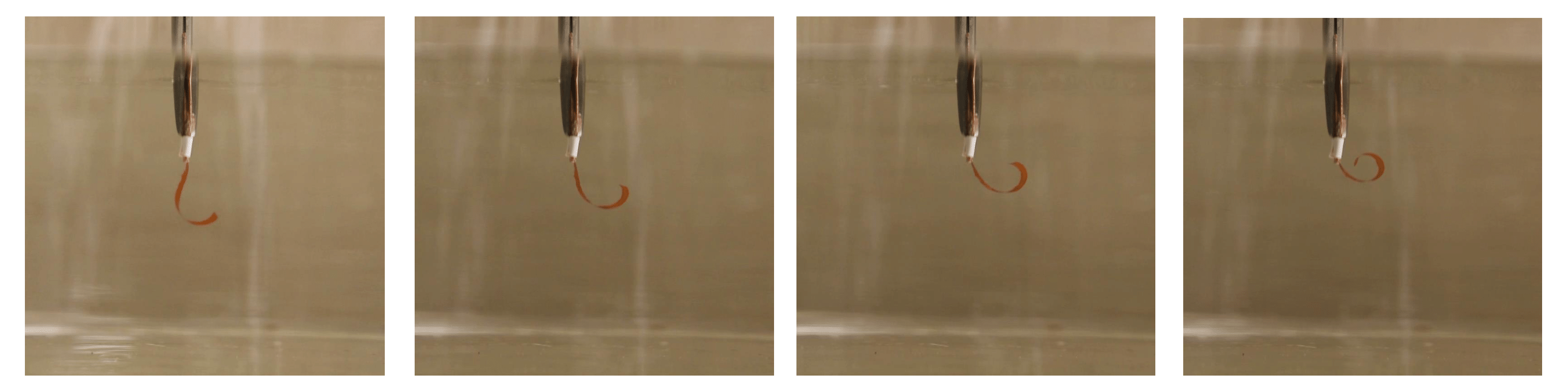


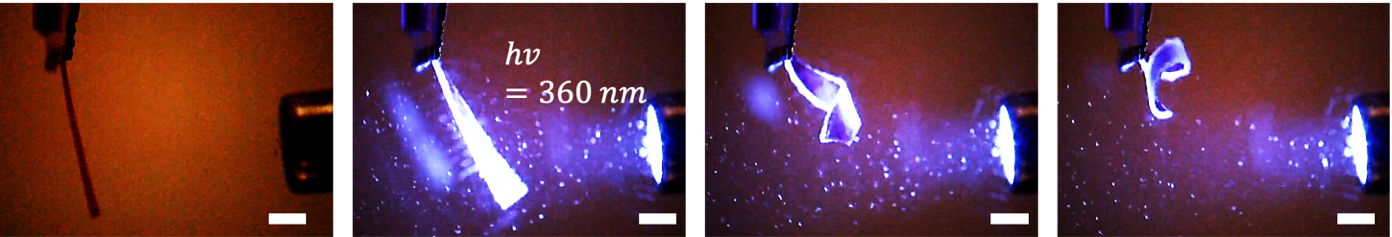


**Figure S8:** Single liquid crystal elastomer thermal actuator in the absence of the liquid crystal hydrogel. Actuation was achieved by thermal actuation in an underwater environment at 55-65ºC in a time lapse 20 seconds*.*

Time-lapse images of the actuation process of the robot with 45º misalignments between the director field of the liquid crystal elastomer and the lyotropic chromonic liquid crystal templated acrylamide/polyethyleneglycol diacrylate gel. Actuation was achieved after irradiation with UV light at 360 nm. Scale bar: 5 mm. The time-lapse between images corresponds to 10 seconds.


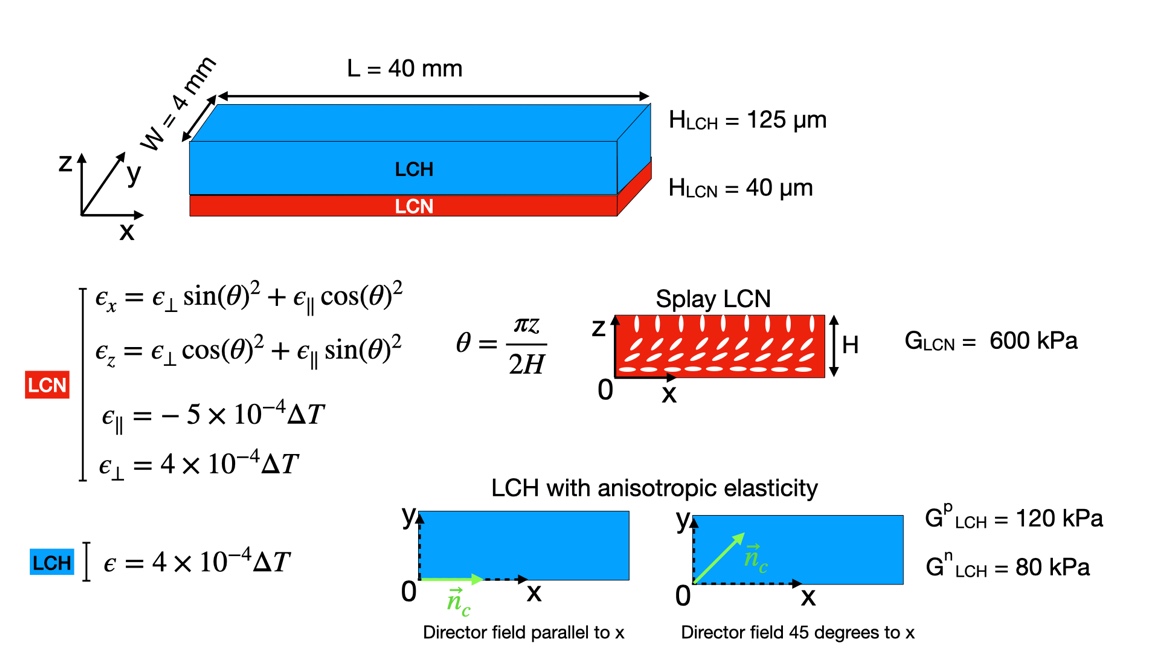


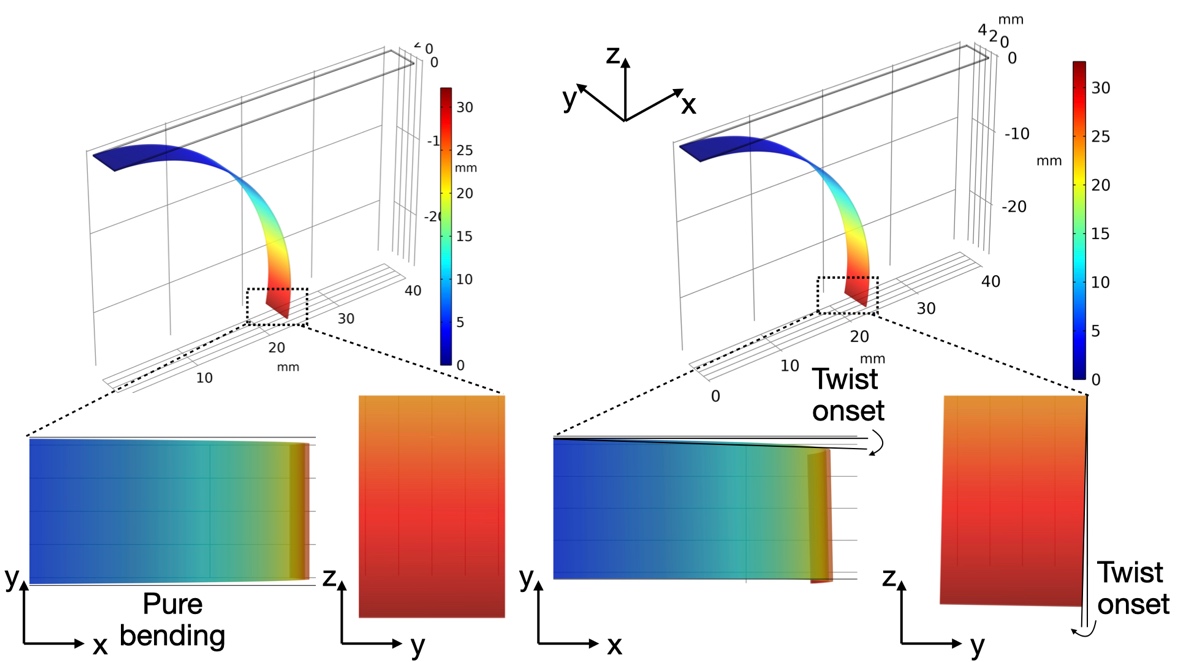


**Figure S9:** FEM modeling of LCH-LCN bilayers; to model the bilayer LCN-LCH structure, we used finite-element simulations in COMSOL Multiphysics 6.3. Using the nonlinear hyperelastic mechanical model with thermal-induced strains, we have performed quasi-static large deformation studies. The mechanical properties of the LCN were modeled using Neo-Hookean model; whereas, the anisotropic mechanical properties of the LCH were modeled using Fung model. For the thermal strain, we have defined the anisotropic thermal expansion/contraction for the splay LCN, as shown in top panel; whereas, for the LCH, we have assumed an isotropic thermal expansion. The bottom panel shows the bending and twisting deformation for the two different configurations: 1) The director of LCH parallel to x-axis, leading to bending; and 2) The director of LCH has 45 degrees rotation in x-y plane, leading to twisting deformation. We should note that due to instability of the simulations during the twist of the beam, we presented the maximum stable solution, to prove the onset of twisting deformation.


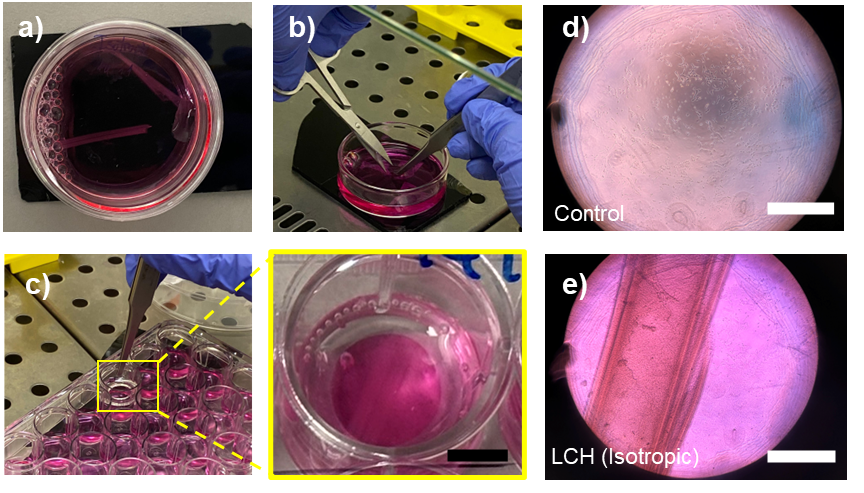


**Figure S10.** Membranes were washed for 48h in sterile MQ-water followed by 24h in complete growth media to remove any non-polymerized material, before being cut (b) and added to the fibroblast cells (3T3), held in contact with them using a PDMS ring (c). d) Inverted microscope images of control and LCH (isotropic) conditions. Scale bar: 5 mm


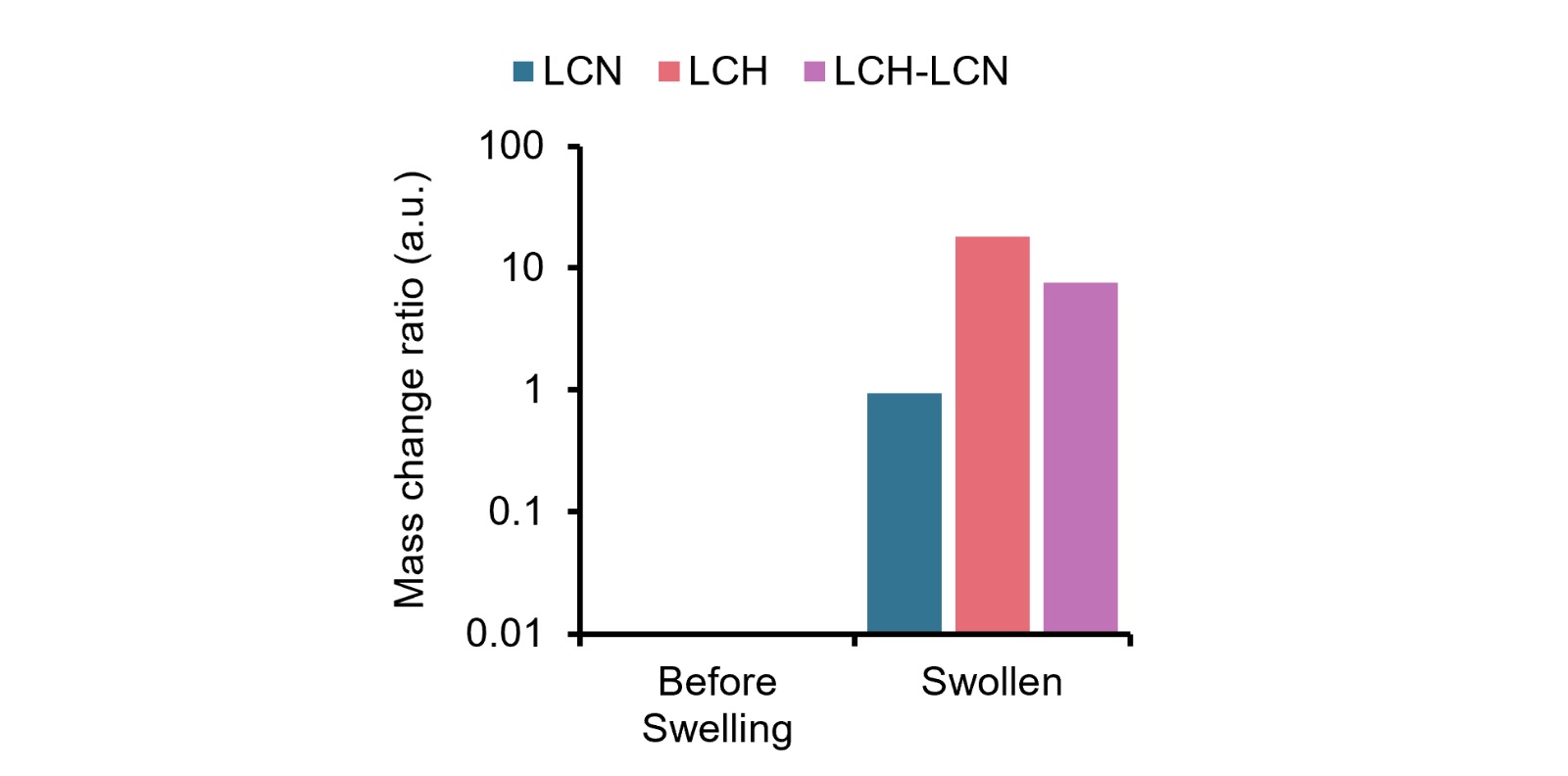


**Figure S11.** Swelling of the LCN, LCH, and bilayer LCH-LCN. Measured by the mass change ratio.

**Supplementary Note 5: Swelling experiments**

We followed a drying and swelling approach in order to load the drug into the bilayer and films. The loading process revealed a clear influence of the LCH component. As shown in **Figure S11**, incorporation of LCH led to a substantial increase in mass, nearly an order of magnitude higher than LCN alone, underscoring its role in enhancing drug uptake. Although the thinner LCH layer in the bilayer resulted in slightly lower loading values compared to the reference hydrogel, its presence nonetheless improved the loading capacity relative to bare LCE. We followed a drying and swelling approach in order to load the drug into the bilayer and films. The loading process revealed a clear influence of the LCH component. As shown in **Figure S11**, incorporation of LCH led to a substantial increase in mass, nearly an order of magnitude higher than LCN alone, underscoring its role in enhancing drug uptake. Although the thinner LCH layer in the bilayer resulted in slightly lower loading values compared to the reference hydrogel, its presence nonetheless improved the loading capacity relative to bare LCE


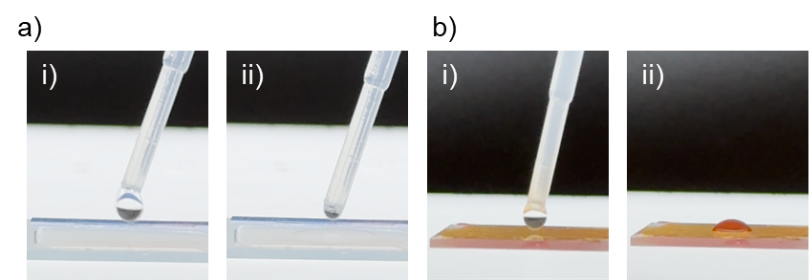


**Figure S12.** Images of the contact angle test on a LCH substrate (a) and on a LCN substrate (b).


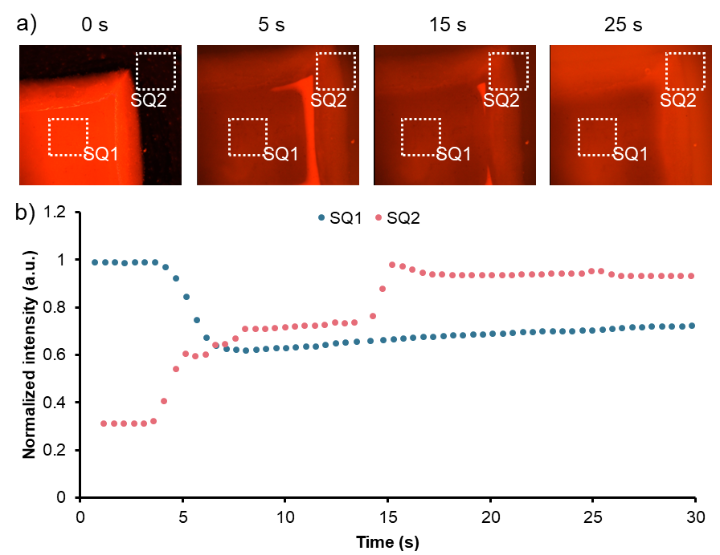


**Figure S13.** a) Sequence of fluorescence microscopy images of DOX release from an LCH sample. b) Normalized intensity measurement of the for DOX release or fluorescence within the sample (SQ1) and outside of it (SQ2).


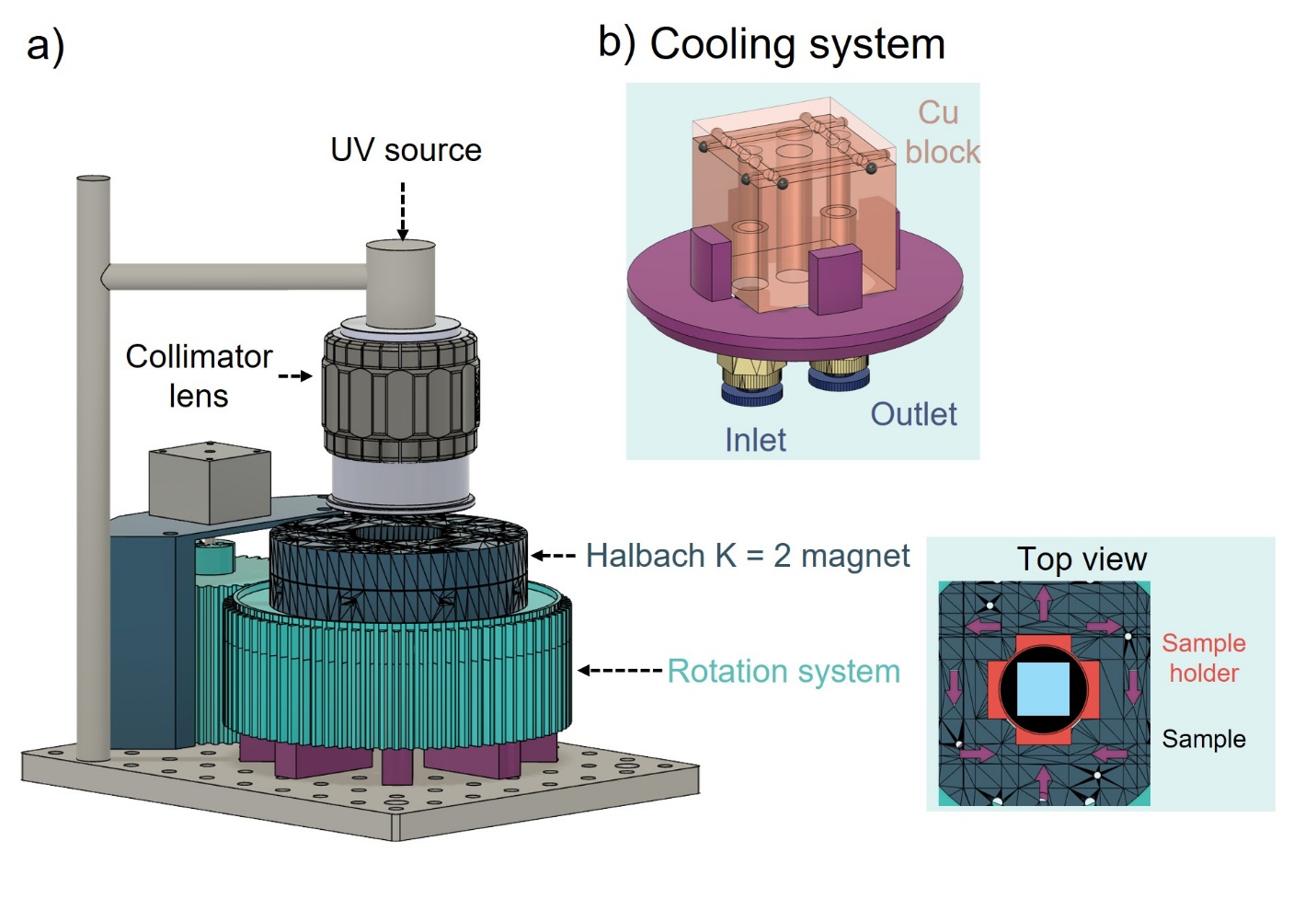


**Figure S14.** a) Schematic representation of the photopolymerization system and b) the cooling system for producing aligned LCLC templated hydrogels.
